# Carbon Monoxide as a Potential Therapeutic Agent: A Molecular Analysis of Its Safety Profiles

**DOI:** 10.1021/acs.jmedchem.4c00823

**Published:** 2024-06-12

**Authors:** Shubham Bansal, Dongning Liu, Qiyue Mao, Nicola Bauer, Binghe Wang

**Affiliations:** Department of Chemistry and the Center for Diagnostics and Therapeutics, Georgia State University, Atlanta, Georgia 30303, United States

## Abstract

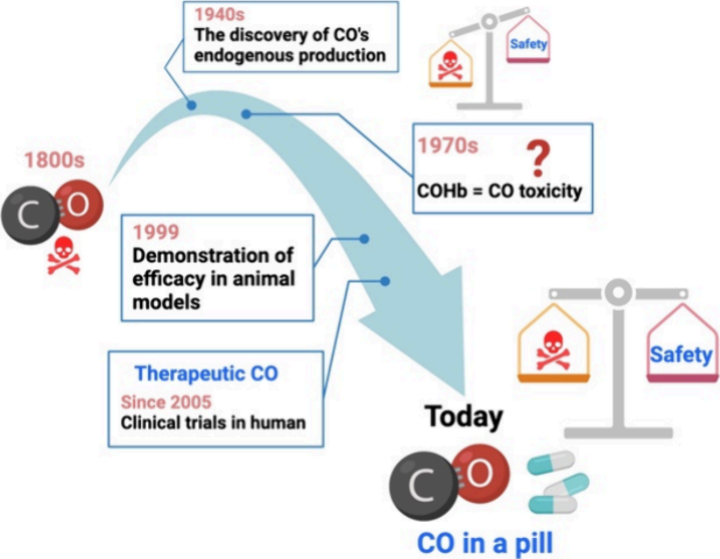

Carbon monoxide (CO) is endogenously produced in mammals, with blood concentrations in the high micromolar range in the hemoglobin-bound form. Further, CO has shown therapeutic effects in various animal models. Despite its reputation as a poisonous gas at high concentrations, we show that CO should have a wide enough safety margin for therapeutic applications. The analysis considers a large number of factors including levels of endogenous CO, its safety margin in comparison to commonly encountered biomolecules or drugs, anticipated enhanced safety profiles when delivered via a noninhalation mode, and the large amount of safety data from human clinical trials. It should be emphasized that having a wide enough safety margin for therapeutic use does not mean that it is benign or safe to the general public, even at low doses. We defer the latter to public health experts. Importantly, this Perspective is written for drug discovery professionals and not the general public.

## Significance

This Perspective highlights the therapeutic potential of CO in areas including organ protection, anti-inflammation, and cancer, among others; shows the lack of toxicity of CO in the high micromolar range in the hemoglobin-bound form (COHb); shows CO’s high safety margin comparable to many approved drugs and nutrients in the body; demonstrates the improved safety margin when delivered via the non-inhalation form; and provides molecular explanations for why the same COHb level may lead to different toxicities.

## 1 Introduction

Carbon monoxide (CO) is produced endogenously through heme degradation by heme oxygenase (HMOX), leading to physiological blood concentrations in the high micromolar range in the hemoglobin-bound form known as COHb.^1,2^ At this point, there is a need for clarification. Despite the conventional use of “carboxyhemoglobin” to refer to COHb, the formal name should be “carbonylhemoglobin” according to the rules of IUPAC nomenclature of chemistry.^3^ With this clarification, we note that the conventional name of “carboxyhemoglobin” (technically incorrect) and the formal name “carbonylhemoglobin” have the same abbreviation of COHb and refer to the same chemical entity.

Recent years have witnessed a steady increase in interest in the signaling roles and therapeutic effects of CO.^2,4^ Studies in animal models have demonstrated the promise of using CO for treating inflammation of various types,^5^ sickle cell disease,^6^ cancer,^7^ and cancer metastasis.^8^ CO has also been shown to offer cytoprotection and organ protection,^9−13^ play an important role in neuromodulation and cognition via a possible CO–dopamine–heme oxygenase signaling axis,^14^ and regulate the circadian clock,^15^ which is further implicated in an array of pathophysiological and pharmacological events.^16^ Despite all the success in demonstrating the pharmacological effects of CO in cell culture and in animal models, there are also many unanswered questions, including issues of sufficiency of the heme supply to allow CO a role in rapid signaling,^17^ the molecular mechanisms(s) of action,^18,19^ and safety. This review focuses on the last question: is CO safe enough to be used for therapeutic applications? Although this question is universally true for any new therapeutic agents, there is an added layer of importance in addressing this question for CO because of the common perception of CO being a poisonous gas regardless of the concentration. Part of this perception issue is rooted in the fact that almost everyone learned about CO for the first time in the context of it being a poisonous gas. Less known is the fact that CO is produced endogenously as part of a normal physiological process in red blood cell turnover through heme degradation by heme oxygenase (inducible HMOX-1 and constitutive HMOX-2), with the production of CO being an obligatory process. The daily “average production” of CO is about 400 μmol per person, leading to the formation of CO-bound hemoglobin, known as COHb. As a result, 2% COHb is considered physiological.^20^ Given hemoglobin’s concentration of about 7.5 mM, 2% COHb corresponds to about 150 μM, which is higher than or comparable to the peak concentrations of many commonly used medications. For example, peak plasma concentrations are 132 μM (20 mg/kg) for acetaminophen,^21^ >300 μM for naproxen,^22^ 1.15 μM (630 ng/mL) for doxorubicin,^23^ 3.1 μM (135 mg/m^2^) for Taxol,^24^ 19.9 μM for cisplatin (1 h infusion),^25^ 3.8 μM (1.26 mg/L) for ciprofloxacin (single oral doses of 250 mg),^26^ and ∼420 μM for 5-fluorouracil (<500 mg dosing).^27^***A safe assumption is that physiological concentrations of CO do not present toxic effects.*** Therefore, the above comparisons lead to two fairly safe conclusions. First, at high micromolar concentrations (e.g., 150 μM), CO in the form of COHb is safe. Second, at concentrations comparable to those of the peak concentrations of many commonly used drugs, CO does not have toxicity issues. Thus, CO is toxic only at high levels. In this review, we hope to present a thorough analysis of CO’s safety profiles and the toxicity issues of CO based on experimental evidence. We hope to show that CO has a high enough safety margin for potential therapeutic use. Here, it is very important to make one point: there is a fundamental difference between using a chemical entity (i.e., a drug) for therapeutic applications compared to analyzing its tolerable level as a pollutant/contaminant. A case in point is the use of antibiotics for treating bacterial infection or an antineoplastic agent for cancer as compared to permissible exposure to the general public as a contaminant.^28−30^ The “cost-benefit” analysis is fundamentally different for these two scenarios. The former is in reference to treating an otherwise harmful condition, and the latter is in the context of exposure levels to an otherwise healthy population with no need for that particular drug. With this preamble, below we provide detailed analyses of CO’s safety profiles in terms of boundary conditions, not as specific guidelines. In doing so, we first present an overall landscape and lay out the organization of the experimental evidence. Then, we present technical details in the various subsequent sections. We intentionally built in some redundancy among the various sections. This is to allow each section to be somewhat “stand alone” and avoid the need for readers to go back and forth in order to understand the materials in a given section.

## 2 An Overview of CO’s Safety Profiles

In this review, we analyze CO’s safety profiles from 10 areas. **First**, CO is produced endogenously as part of a normal physiological process in red blood cell turnover as described earlier.^20^ There is a small portion of CO that comes from the metabolism of heme sourced from other hemoproteins such as cytochrome P450. There is also the possibility of CO coming from the gut microbiome.^20^ CO largely exists in the form of COHb at varying levels under normal physiological conditions. Depending on which publications to read, 1–2% COHb (corresponding to 75–150 μM) is considered physiological. All this means that medium to high micromolar concentrations of CO are endogenous in the human body, physiological, and do not seem to be toxic. **Second**, there have been numerous human clinical trials examining CO’s safety, leading to the conclusion that CO at up to 6.4% COHb is considered safe for these trials.^31^ In one case of kidney transplantation studies, 14% COHb was set as the threshold for clinical trials using inhaled CO.^32^ Such studies demonstrate that CO is safe enough for therapeutic assessments at a concentration many-fold higher than the commonly accepted physiological levels. It is important to note that 10% COHb corresponds to about 750 μM, which is probably higher than the therapeutic concentration of most FDA-approved drugs. It is also important to point out that efficacious levels of CO in animal models fall within this range. **Third**, CO has a safety margin comparable to or higher than those of many commonly encountered drugs or endogenous biomolecules. **Fourth,** there is extensive literature evidence to show that CO delivered via a noninhalation form is much safer as compared with inhaled CO.^19,33^ For example, in a famous dog experiment by Goldbaum, COHb levels reached 55%, 70%, and 80% 2 h after the administration of 50, 150, and 200 mL/kg pure CO gas, respectively, via intraperitoneal injection (i.p.) and returned to normal within 24 h.^34^ During the experiments, no change in appetite or behavior of the dogs was observed.^34^ In contrast, comparable COHb levels are almost certainly associated with death when CO exposure occurs via inhalation.^19,35−38^ All these lead to the **fifth** point, “Despite that blood COHb levels are commonly measured in patients with CO poisoning, the clinical presentation often does not correlate with the COHb level.”^39^ In other words, COHb should not be regarded as the single indicator of CO exposure in the context of the safety of CO. Instead of COHb level alone, a second relevant parameter is probably tissue concentration or more precisely the engagement of critical targets in tissue, which is a complex issue that needs to be examined extensively.^19^ Furthermore, because hemoglobin is a tetramer, COHb can exist in many forms (i.e., Hb_4_(CO)_4_, Hb_4_(CO)_3_(O_2_), Hb_4_(CO)_2_(O_2_)_2_, and Hb_4_(CO)(O_2_)_3_). At the same level of COHb%, variations in the composition of these four forms can make a fundamental difference in terms of toxicity. This point is analyzed in depth later. Therefore, the known safety issues with CO at a certain level of COHb resulting from CO inhalation may or may not be an issue if CO is administered via a noninhalation route. This is also the reason that we have devoted extensive efforts to developing “CO in a pill” and understanding target engagement, pharmacokinetic (PK) issues, and tissue distribution.^19,33,40^**Sixth**, at the molecular level, a complex array of factors affects the differential CO toxicity (inhalation vs gastrointestinal (GI)/systemic delivery), including the following: binding kinetics; the varying affinity of hemoglobin for CO depending on pH, O_2_ contents, and other chemicals such as 2,3-diphosphoglycerate (2,3-DPG); and the relative binding affinity of various hemoprotein targets, including hemoglobin and myoglobin. There have been extensive analyses of these issues.^19,33−38,41^ For example, because hemoglobin largely exists in the high-affinity R-state in the lung and the low-affinity T-state in peripheral tissues, the ratio of the affinity of hemoglobin for CO/O_2_ is estimated to be about 180 in the lung (R-state) and 390 in peripheral tissues (T-state). As such, there are good reasons to believe that the composition of the four forms of COHb at the same COHb level can be different depending on the site of CO delivery/binding; inhalation delivery of CO leaves much “free CO” in the lung to travel with the blood, diffuse, and engage targets in vital organs, whereas systemic or oral delivery may allow CO to rapidly bind to hemoglobin but not to engage with targets in vital organs to the same extent.^19,33,41,42^ Therefore, noninhalation delivery is expected to offer improved safety and efficacy profiles compared to inhaled CO. Indeed, efficacies of CO below 6% COHb have been reported when CO prodrugs are used in various animal models.^33,43,44^**Seventh**, all this points to enhanced safety profiles of CO delivered when using a CO donor, which has the likelihood of offering efficacy at a safe level of COHb. **Eighth**, the concept of considering the potentially negative impact of a chemical entity as an environmental pollutant or a contaminant is different from that entity being a therapeutic agent. Indeed, antibiotics as pollutants/contaminants in drinking water or milk and their potential harm to a healthy population^45^ are different questions from the use of antibiotics for treating bacterial infection. In the latter case, it is the safety margin that matters for all therapeutics. Along this line, absent the need to use CO to treat a harmful condition, the safety issue of using CO as a pollutant or as a byproduct needs to be examined in a different context. There is a large body of literature for readers to understand the subject of CO as a pollutant.^46−49^ Again, CO is acutely poisonous at high levels. However, for CO to be used as a therapeutic agent, all the available indications are that it has a sufficiently high theoretical safety margin, which is comparable to or higher than those of other commonly seen drugs and endogenous biomolecules such as insulin, potassium, doxorubicin, digitalis, and warfarin, among many others.^42,50^ A detailed analysis is provided in subsequent sections. **Ninth**, targeted delivery and new formulations could offer improved safety profiles.^51−55^ We believe that CO delivered in the form of a prodrug is expected to offer a sufficient safety margin for therapeutic use, as has been demonstrated in a large number of studies in animal models of cancer, organ injury, and inflammation.^43,55−58^**Tenth**, the reversible nature of binding of CO to a hemoprotein and the endogenous nature of CO mean that long-term exposure to a low level of CO is not expected to have “cumulative effects” or “chronic effects” the same way as in the case of heavy metals, which accumulate in the body, and alkylating agents, which have long-lasting effects. Further, CO overdose can be reversed by oxygen. With “CO in a pill”, overdose is far less likely to be a problem compared to using inhaled CO. Below, we provide detailed discussions of all 10 points by analyzing experimental results and interpretations from published literature.

## 3 Endogenous Production of CO

In assessing the safety profiles of CO, it is important to keep in mind its endogenous production under normal physiological conditions. Such endogenous production already strongly suggests that CO is not toxic unless it is beyond physiological levels. The endogenous production further suggests a possible regulatory role for CO in normal pathophysiological processes. In this section, we briefly discuss CO in terms of its production process, its storage and transport within the human body, physiological concentrations, its engagement with various molecular targets, and concentrations under various pathological conditions. Such combined information should lay a general framework of acceptable therapeutic levels of CO in different contexts as well as levels frequently seen under various pathological conditions.

**Figure 1:**
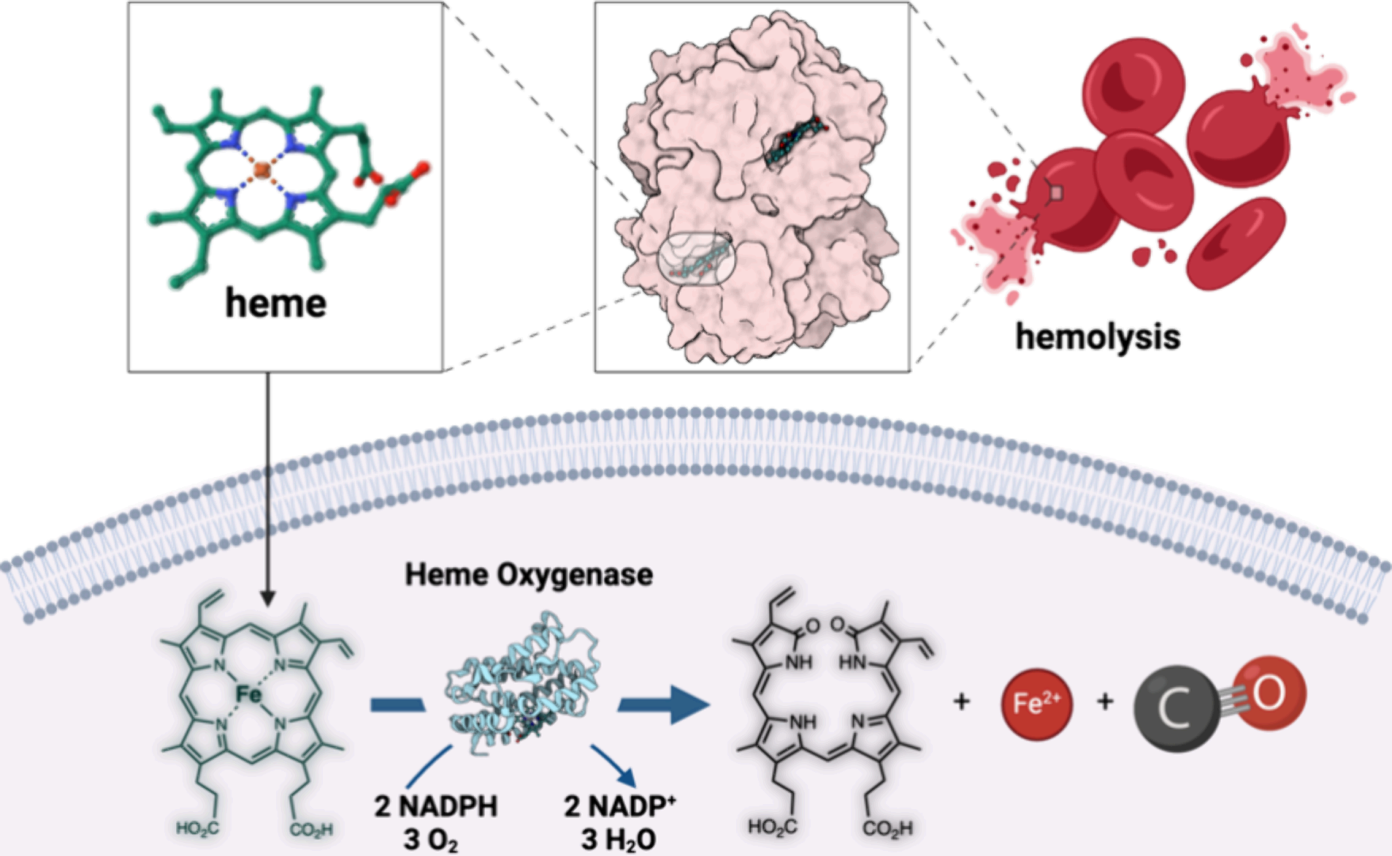
Endogenous production of CO. The major endogenous source of CO is heme degradation by heme oxygenase. Figure adapted from images created with BioRender.com.

Briefly, as early as the 1940s, work by Sjostrand led to the discovery of the endogenous production of CO.^59^ The major source of CO was later (1957) characterized by Ludwig and co-workers to be through heme degradation by HMOX,^1^ which has two isoforms: inducible HMOX-1 and constitutive HMOX-2 (Figure 1).^60,61^ The major source of heme is red blood cells. Given the fact that red blood cells turn over three times a year, CO production is substantial. The “average” rate has been estimated to be 18.8 μmol/h (0.42 mL/h), which gives about 450 μmol (12.6 mg) per day, leading a “normal” COHb level of 0.3–1%.^62^ Of course, substantial individual variations are expected, as discussed below. Nevertheless, this approximate number provides a reference point. CO has limited solubility at about 1 mM under 1 atm of CO.^63^ The CO concentration in the air is normally in the range of 0.5–5 ppm in a household (with the dissolved concentration estimated to be 0.5–5 nM in solution using Henry’s law). In the vicinity of a properly adjusted gas stove, the CO level can be up to 5–15 ppm; in the vicinity of a poorly adjusted gas stove, the CO level can be up to 30 ppm according to the U.S. Environmental Protection Agency (EPA).^64^ CO largely exists in the form of COHb. The presence of up to 2% COHb is considered physiological, corresponding to about 150 μM when calculated using the average hemoglobin level of 7.5 mM.^20^ This means that under normal physiological conditions CO is largely stored in a hemoglobin “reservoir” as COHb. At this point, it is important to note that, similar to its binding to oxygen, hemoglobin binds with CO with varying affinity depending on other factors. Briefly, hemoglobin has two affinity states: the high-affinity R state and the low-affinity T state.^65^ The former exists in well-oxygenated environments at high pH (e.g., arteries and the lungs), and the latter exists in poorly oxygenated environments and at low pH (e.g., veins and peripheral tissues).^66^ The affinity of hemoglobin for CO is between a dissociation constant (*K*_d_) of 0.7–1.7 nM in the R state and that of about 1.1 μM in the T state.^33^ The varying affinity for CO (and O_2_) is very important for target engagement (and O_2_ delivery) and CO toxicity analysis, which are further discussed in a later section.

In addition to the accepted COHb levels (<2%) under physiological conditions, it is important to also discuss variations in COHb concentrations within the general population, including pregnant women, infants, and those with hematological diseases. In a later section, CO exposure in smokers is discussed. Below, we discuss the details.

First, the rate for CO production varies among organs and tissues; under normal physiological conditions in a healthy population, there are significant variations in the COHb level and CO production rate. An excellent review on this subject by Owens was published in 2010.^62^ Briefly, the major organ for CO production is the liver, followed by the spleen, brain, and erythropoietic system. These are also among the most metabolically active sites. As discussed earlier, the daily production of CO gives a “normal” COHb level of 0.3–1%. In other studies, using donated blood, the average COHb level was determined by a blood gas analyzer to be 0.78% with a standard deviation of 1.48%, with the maximum being 12%.^67^ It is important to note that there are other normal physiological conditions that lead to variations in this “normal” COHb level. For example, women experience substantial fluctuations in COHb level throughout menstruation, with endogenous CO production doubling in the progesterone phase (0.62 mL/h vs 0.32 mL/h in the estrogen phase).^68,69^ Further, endogenous CO production increases during pregnancy (0.92 mL/h).^69,70^ Neonates and infants also have an elevated level of CO production. For example, rates of CO production are reported to be 13.7 ± 3.6 μL/kg/h in infants^71^ and 6.1 ± 1.0 μL/kg/h in men.^70^ Incidentally, CO production seems to be different between men and women (5.24 ± 0.66 μL/kg/h in the estrogen phase and 10.2 ± 0.99 μL/kg/h in the progesterone phase^68^), with enhanced CO production in the progesterone phase possibly due to the effect of heme synthesis induced by progesterone.^72^ Exercise also increases the COHb level in healthy humans.^73^ For example, ten nonsmoker healthy volunteers (five male and five female) were involved in a study. After the volunteers exercised on the bicycle ergometer for 15 min and then rested for 15 min, COHb level doubled (1.1 ± 1.6 to 2.1 ± 1.6%), only returning to the initial level (1.3 ± 1.3%) after 1 day.^73^ Additionally, trace amounts of carbon monoxide are also produced in the intestines through the gut microbiome.^74^

As discussed earlier, endogenous CO production is associated with heme oxygenase activity and hemolysis. Thus, any disease with hemolysis as a component may lead to changes in endogenous CO production such as sepsis, sickle-cell anemia, and other hemolytic diseases. Endogenous CO production is elevated in patients who require mechanical ventilation with severe sepsis compared with ICU controls (10.9 ± 5 μL/kg/h on day 1 vs 2.1 ± 0.5 μL/kg/h ICU controls).^75^ Moreover, exhaled CO concentrations in sepsis patients were found to be significantly higher than ICU controls (1.53 ± 0.42 ppm on day 1 vs 0.54 ± 0.09 ppm ICU controls).^75^ Higher COHb levels were detected in sickle-cell disease patients (6.46% for smokers and 4.29% for nonsmokers)^76^ relative to the normal level (<2%). The end tidal CO (ETCO) values of hemolytic neonates were found to be significantly higher (7.3 ± 0.6 ppm) compared with those of healthy term nonhemolytic neonates (1.9 ± 0.6 ppm).^77^ Moreover, higher exhaled CO levels were also confirmed in children with sickle cell disease.^78^

Specifically, a 2006 study reported the CO level in the intestinal lumen of patients with ulcerative colitis to be around twofold higher than that of healthy volunteers (1.0 ppm ± 0.19 ppm vs 0.45 ± 0.04 ppm).^79^ Moreover, the elevated CO level is consistent with the increased expression of heme oxygenase in the mononuclear cells at the site of intestinal inflammation.^79^ In another study in 2016, Roy et al. reported that the CO content in the flatulence of patients with gastrointestinal disease was 20× higher than that for healthy volunteers (258 ppm vs 10–13 ppm).^80^

There are additional factors to consider when discussing normal CO production.^20^ Catabolism of heme from nonhemoglobin sources is a minor component but an important consideration.^81^ CO is largely exhaled intact. However, some oxidation (<10% in dog experiments) by cytochrome *c* oxidase could happen in the mitochondria, leading to CO_2_ production. To add to the complexity of the issue, increased CO production is not always correlated with increased COHb levels.^62^

In addition to CO production via heme degradation under various pathophysiological conditions, there is another very interesting angle that ties the concentration of “free CO” with exposure to light and then possibly circadian controls. In 1996, Oren proposed a theory of humoral transduction, where due to structural similarities between chlorophyll chromophores and heme moieties the components in blood may act as messengers.^82^ Specifically, it was proposed that “light-driven retinal synthesis and release of neuroactive gases, such as CO and NO, and consequent stimulation of blood flow transmit to the brain a signal of day”.^82^ Basically, CO produced in the retina could allow direct access of the gasotransmitter to the brain. Of course, there is a distinction between CO release and CO synthesis. However, the end results are similar, i.e., an increase in CO concentration in the free form to engage a target.^19^

**Figure 2:**
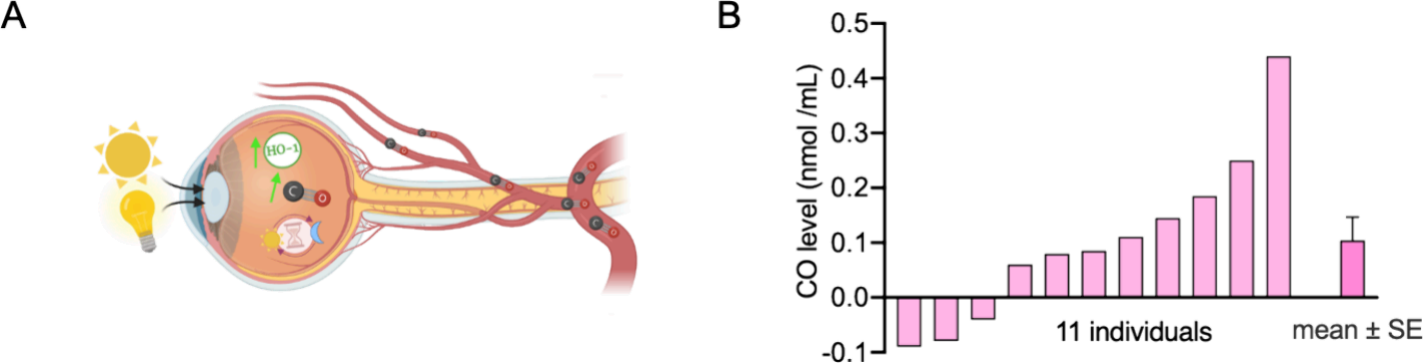
CO released into the ophthalmic venous blood (OphVB) depending on the intensity of sunlight. (A) Light triggered increase in endogenous CO. (B) The change in CO levels (mmol/mL) in venous blood of 11 individual pigs after 80 min of 5000 lx white light exposure. Adapted from ref (84). Copyright 2017 Elsevier. Figure adapted from images created with BioRender.com.

Interestingly, CO has been reported to be endogenously released into ophthalmic venous blood (OphVB) depending on the intensity of sunlight (Figure 2A). In a study by Koziorowski and co-workers, the concentration of CO increased threefold in OphVB during the longest days of summer in comparison to the shortest days of winter (3.43 ± 0.8 nmol/mL and 1.11 ± 0.10 nmol/mL, respectively).^83^ Along the same line, a study by Oren and co-workers reported a 25% increase of the mean CO concentration found in the retinal venous blood from the ophthalmic sinus (mean increase of 0.10 nmol/mL ± 0.05) in male pigs that were exposed to 5000 lx white light (Figure 2B).^84^ There are two possible mechanistic explanations for this increase in endogenous CO, one of which dates back over a century. In 1896, Haldane and Smith reported that, through varying seasons and hours, ambient light could break the bond between CO-Hb.^85^ These results were dismissed due to the purported lack of physiological relevance of CO at the time, when only one metal complex (Ni(CO)_4_) had been identified and the concept of its photodissociation was unknown.^86^ It is now well-understood that metal carbonyl complexes release CO via photoactivation.^87,88^ Therefore, it is plausible that the increase in the CO concentration is sourced from the photodissociation of CO from CO-Hb. Another possible explanation came in 1996, when Kutty and co-workers reported that intense visible light induced HMOX-1 in the retina.^89^ Interestingly, Oren and co-workers also proposed a role for bile pigments, such as bilirubin, in sleep and regulation of the biological clock.^90^ Because bilirubin is a product of HMOX-1 and has been reported to be a signaling molecule,^91^ it is plausible that light-induced expression of HMOX-1 is a mechanism for increasing the endogenous CO concentration, which could exert various signaling functions synergistically and/or cooperatively with bilirubin. Both mechanisms support the ability to increase the concentration of CO through light triggers. The catalytic release of CO from hemoglobin by light was further confirmed in an *in vitro* study conducted by Oren and co-workers in 2020.^92^ In this study, blood samples were taken from two sample sets of women (*n* = 24 and *n* = 11) and exposed to 2 h of bright white light (10 000 lx). The “free CO” produced was determined by using GC-FID headspace quantification. The authors found that the concentration of ambient CO doubled after the samples were exposed to 2 h of bright light. Interestingly, in a posthoc analysis, an inverse correlation between the time of day and the CO concentration was seen in a statistically significant manner in the first study (*n* = 24) and a nonstatistical manner in the second study (*n* = 11). Specifically, samples taken after 8 AM showed a time-dependent decrease in the degree of ambient CO when exposed to 2 h of bright light.

Although much more work needs to be done to determine if CO is a transmitter of a light signal, there is sufficient evidence to support an increase in free CO during light exposure. These results suggest there are potential roles for CO in many different areas where light response is key, such as the circadian rhythm and possibly cognition.^14,93^

Overall, there is a significant level of CO that is intrinsic to the human body. The general population without pathological conditions routinely experiences medium to high micromolar concentrations of CO in the form of COHb. The correlation of CO production changes with certain physiological processes such as menstruation and physical exercise indicates some regulatory/signaling roles for CO. There is also evidence to suggest a role for CO in circadian rhythm. With all that said, there are more unknowns regarding the molecular mechanism(s) of actions of CO, whether there is a sufficient supply of heme to allow CO a role in rapid signaling in the same way as known second messengers such as cA(G)MP and Ca^2+^, what factors affect the correlation of CO production and COHb level, and what level of CO is considered toxic or lethal. Much more work is needed to fully understand the biological roles of this small molecule.

### 3.1 Smoking and COHb Levels

In examining the issue of CO exposure levels in the general population, we would be remiss if we did not discuss the smokers’ population and second-hand smoking. Because of the widely recognized harmful effects and the significant public health consequences of smoking, there have been extensive efforts to study the contents of harmful chemicals in cigarette smoke.^94^ There are hundreds, if not more, known harmful chemicals in cigarette smoke, including aldehydes, ethylene epoxide, acrylamide, acrolein, pyridine, aniline, acrylonitrile, nitro compounds, nitroso compounds, and hydrazine. Among them, a large number (80+) of such chemicals are classified as carcinogens. Though not classified in this long list of carcinogens, CO is commonly used as a marker in studying the smoking exposure level. This is easy to understand because CO is probably the easiest to measure among the large number of chemicals in cigarette smoke and is one of the few that is somewhat quantitatively proportional to the act of smoking regardless of the brands. Further, CO is recognized as a contributing factor to the development of cardiovascular disease (CVD), largely because of its ability to bind to hemoglobin and thus impact the ability of hemoglobin to deliver a healthy dose of oxygen to tissues. Because of the extraordinarily large number of publications in smoking and CO, we select two government reports as lead references and a window to publications in this regard.^95,96^ We defer to public health experts and these government reports on all public-health-related implications of CO exposure. In this Perspective, we focus on examining the COHb levels commonly seen in smokers or second-hand smokers and on issues at the molecular level. Further, the issue of CO-Hb binding is discussed in detail in section 5.

The COHb level is greatly influenced by smoking or second-hand smoke. Based on careful determination of CO yields in the mainstream smoke of selected international brands of cigarettes, smoking has been reported to deliver 5.9–17.4 mg CO per cigarette.^97^ This is roughly on the scale of the daily endogenous production of CO by an “average” person. Regular smokers have COHb levels ranging around 3–8% compared with <2% COHb in nonsmokers. For example, in the Renfrew/Paisley study in Scotland, the relationship between COHb concentration and the number of cigarettes smoked a day was examined,^62,98^ leading to some quantitative correlations (the mean (standard deviation) COHb level: nonsmoker, 1.59% (1.72); 1–5 cigarettes/day, 2.31% (1.94); 6–14 cigarettes/day, 4.39% (2.48); 15–24 cigarettes/day, 5.68% (2.64); and >25 cigarettes/day, 6.02% (2.86)). In another study of 11 403 men aged 35–64 years,^99^ an approximate linear relationship between COHb and the number of cigarettes smoked was reported (the mean COHb level: nonsmoker, 0.79%; 1–9 cigarettes/day, 1.74%, 10–14 cigarettes/day, 3.07%; 15–19 cigarettes/day, 4.09%; and 20 cigarettes/day, 4.77%). The COHb level was up to 6.54% for those who smoked >40 cigarettes/day. Moreover, in a single case, the COHb level in one patient reached as high as 38.6% through frequent smoking of hand-rolled newspaper cigars.^100^ In a case report of a single waterpipe tobacco smoking session, the loads of CO was quantified as 192 mg (77.5–307 mg) per session, which normally lasts about 45–60 min.^101,102^ Considering its low molecular weight, this is a large amount of CO intake and is roughly 10-fold higher than the endogenous production by an “average” person. In two studies in Saudi Arabia and Pakistan, the COHb level was found to be significantly higher in waterpipe smokers (10.06% and 10.5%, respectively) as compared to cigarettes smokers (6.74% and 6.2%, respectively).^103,104^ In a single patient case, the COHb level reached 39.2% with some symptoms (dizziness and vomiting).^105,106^ Moreover, after smoking a waterpipe for 24.9 min (SD 16.0), smokers exhibited higher COHb levels (presmoking 1.0% (SD 0.4), postsmoking 5.8% (SD 3.7)) when compared to those who smoked a single cigarette for 11.5 min (SD 4.2) (presmoking COHb level of 2.9% (SD 1.4), postsmoking COHb level of 3.7% (SD 1.4)).^107^ With a solubility of about 1 mM, water would stop “filtering out” CO once its concentration reaches 400 nM if the smoke has 400 ppm of CO.^108^ All these results show that smokers have a higher level of exposure to CO and higher COHb level on average than nonsmokers.

The discussions from the above two sections indicate that COHb in humans has a wide range of tolerable levels, which the general population encounters on a routine basis. Though “tolerable levels” generally mean a lack of acutely harmful effects, this does not necessarily mean “non-harmful” levels. A case in point is the high levels of COHb in smokers. Just because this population tolerates a high level of COHb throughout their life, it does not necessarily mean there is no negative effect for the high COHb level. We defer this latter issue to public health experts. In the context of using CO as a potential therapeutic agent, it is a benefit analysis with the understanding that all drugs have some potential health problems at a dose beyond their prescribed level or even at the prescribed level. The potential side effects are analyzed in the context of the severity of the health problems to treat. For example, for treating terminally ill cancer patients, the tolerable side effects are expected to be very different from those for treating chronic muscle aches and pains. The safety issue of using CO needs to be analyzed in the context of a specific disease that it is used to treat. In this Perspective, we only lay out general parameters and ways to examine associated safety issues. ***This is not to assess absolute safety***.

## 4 Clinical Trials in Humans

CO has been studied in a large number of human clinical trials, with the majority intended for safety assessments. There is a recent book chapter on this topic.^109^ In this section, we select a few examples with the aim of discussing boundary conditions demonstrated by these clinical trials in terms of human safety and levels of exposure.

**Figure 3:**
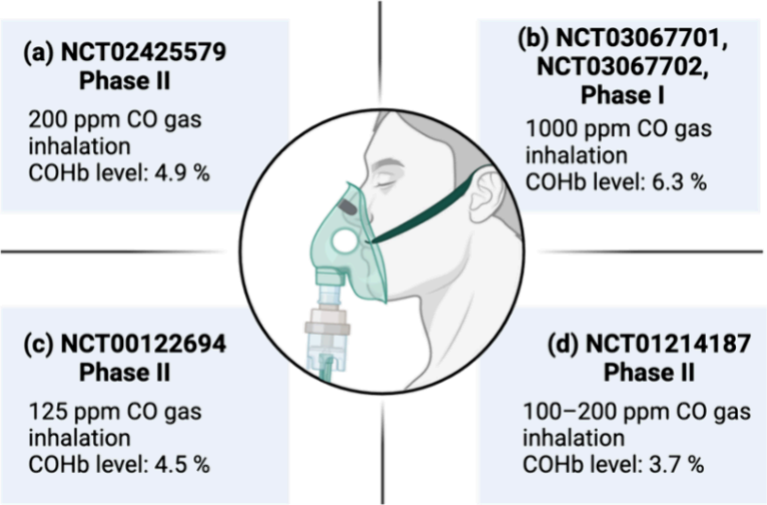
Human clinical trials of inhaled CO gas. CO inhalation conditions are the following: (a) 200 ppm of CO for 90 min/day for 5 days; (b) 1000 ppm of CO for 30 min at the rate of twice every minute, with a 1 min break every 5 min; (c) 125 ppm of CO for 2 h/day for 4 days; and (d) 100–200 ppm of CO for 2 h/day twice a week for 12 weeks. Figure adapted from images created with BioRender.com.

Since 2015, there have been three clinical trials (NCT02425579, NCT03799874, and NCT04870125) on using inhaled carbon monoxide to treat sepsis-induced acute respiratory distress syndrome; two phase 1 trials and one phase 2 trial. One of the clinical trials was completed (NCT02425579) (Figure 3) in 2019:^31^ inhalation of 100–200 ppm of CO gas for 90 min for 5 consecutive days was found to be well-tolerated by 12 participants. As a result of the inhalation, COHb levels increased from 1.97% (placebo air-treated) to 3.48–4.9%. There are no results published for the other two trials. However, these COHb levels are not beyond what has been reported for samples from the general population.

There are also two clinical trials on the effects of CO on blood vessel functions (NCT03067701 and NCT03616002) (Figure 3).^110^ In one case, eight young participants (around 26 years old) were allowed to inhale 1000 ppm of CO (0.1% CO) for 30 min at the rate of twice every minute, with a 1 min break every 5 min. In another group, data from 30 hookah smokers (charcoal-heated) were recorded. COHb levels were found to be similar (6.3%) in these two groups. The participants did not report any adverse effects. Further, flow-mediated dilation (FMD) of the brachial artery was measured as an indication of blood vessel functions. It was found that inhaling CO gas and smoking charcoal-heated hookah significantly increased the FMD level by 138 ± 71% and 43 ± 7%, respectively. As a comparison, a decreased FMD level (by 27 ± 4%) was observed from smoking electronic-heated hookah. These results show that inhaling CO can influence blood vessel functions as a vasodilator.

In 2005, a phase 2 clinical trial (NCT00122694) (Figure 3) used inhaled CO gas to treat chronic inflammation in patients with stable chronic obstructive pulmonary disease.^111^ In a pilot safety study, one healthy subject inhaling 100 ppm of CO gas with a flow of 10 L/min for 75 min achieved a maximal COHb level of 2.7% without any adverse effects. Three patients with stable COPD achieved a COHb level of 3.9% without any adverse effects after inhaling 95 ppm of CO for 2 h per day on 4 consecutive days. In an expanded study, 20 stable COPD patients were allowed to inhale CO 2 h per day for 4 days; 100 ppm of CO led to a median COHb level of 2.6%, with the highest being 3.5%, and 125 ppm of CO led to a median COHb level of 3.1%, with the highest being 4.5%.

In 2010, a phase 2 clinical trial (NCT01214187) (Figure 3) for the treatment of idiopathic pulmonary fibrosis (IPF, 29 participants) also showed toleration of 100–200 ppm of CO for 2 h per day twice per week for 12 weeks, with the COHb level reaching 3.7%.^112^

In conclusion, low doses of inhaled CO (100–250 ppm) have been shown to increase the COHb level up to 4.5%. Although CO clinical trials have not yielded convincing efficacy data for CO as a therapeutic agent, they do provide safety data for inhaled CO gas. Further, hookah smoking and inhaling 1000 ppm of CO for 30 min increase the COHb level to 6.3%. These levels of exposure seem to be well tolerated in human studies.

## 5 CO Has a Comparable or Higher Safety Margin than Some Commonly Known Endogenous Biomolecules and Commonly Used Drugs

**Figure 4:**
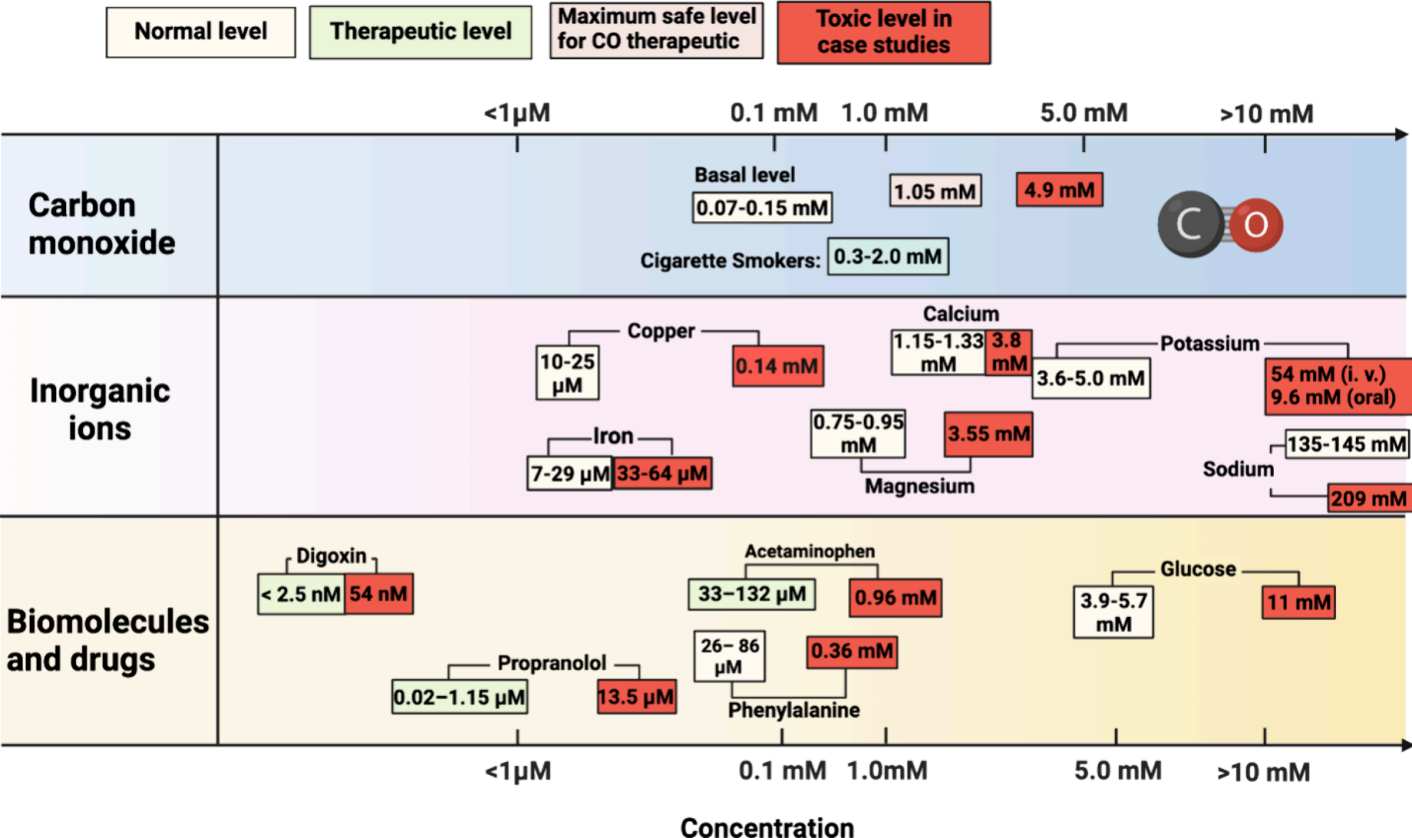
Comparison of CO concentrations under various conditions with commonly known endogenous biomolecules and commonly used drugs. The concentration of CO was converted from COHb%: endogenous COHb level, 1–2% (corresponding to 75–150 μM); cigarette smokers’ COHb level, 4.2–39% (corresponding to 0.3–2.0 mM); COHb safety threshold level in an FDA-approved clinical trial of kidney transplantation, 14% (corresponding to 1.05 mM); and COHb level for imminent threat of death by CO poisoning, 65% (corresponding to 4.9 mM).^113^ The concentrations of inorganic irons, biomolecules, and drugs are shown in Tables 1–3 with references. The toxic levels of inorganic irons, biomolecules, and drugs are based on case studies shown in Tables 1–3 with references. Figure adapted from images created with BioRender.com.

In this section, we examine the safety margins of commonly used drugs and other endogenously produced molecules in comparison with that of CO. Figure 4 shows the comparisons in a graphical fashion, and Tables 1–3 list some specific numbers with references.

**Table 1:** Physiological, Abnormal, and Harmful Levels (Based on Case Studies) of Some Commonly Encountered Biomolecules^a^

#	biomolecules	normal level	abnormal level	harmful level (case studies)	ref
1	glucose	3.9–5.7 mM (fasting)	5.6–6.4 mM prediabetic (fasting), ≥6.5 mM diabetic (fasting)	hyperglycemic hyperosmolar syndrome at 11 mM	(114, 115, 122, 123)
				clinical symptoms include severe dehydration, reduced insulin sensitivity, insidious glucose intolerance, high serum osmolality >320 mOsm/kg, serum bicarbonate >15 mmol/L, pH > 7.3; one case study also reported these symptoms: thirst, vomiting, fatigue, polydipsia, polyuria, Kussmaul breathing, and lethargy	
2	insulin	112 μIU/mL (random)	402 μIU/mL confirms exogenous insulin leading to hypoglycemia as a result of a suicide attempt		(117, 124)
		<25 μIU/mL (fasting)			
		18–276 μIU/mL (1 h after glucose administration)			
3	epinephrine	1.5–3 × 10^–10^ M	chest pain, nausea, and diaphoresis at 1628 ng/L (8.9 nM)		(125−127)
		(0.15–0.3 nM)			
4	phenylalanine	41–68 μM (adults)	121 μM, mild PKU	364 μM, severe PKU	(119, 120)
		26–86 μM (children)			

a The specific values might differ depending on which reference is checked. Abnormal and harmful levels are from case studies and are not meant as guidelines.

**Table 2:** Physiological and Harmful Levels of Select Nutrients^a^

#	nutrients	normal serum concentrations	toxic serum concentrations and effects (case studies)		ref
1	potassium	3.6–5 mM	54 mM^b^	death (IV administration)	(128, 132−134)
			9.6 mM	death (oral administration)	
2	sodium	135–145 mM	190 mM,^c^ 209 mM	death due to ingestion (mistaken for sugar)	(135−138)
3	magnesium	0.75–0.95 mM	3.55 mM	stroke, cerebral metastases, seizure, diminished deep tendon reflexes, progressive muscle weakness including respiratory muscle weakness resulting in respiratory paralysis and coma	(130, 131, 139)
4	copper	10–25 μM^b^^,^^d^	140 μM	renal failure, aspiration pneumonitis, macroscopic hemeaturia, oliguria, and tachycardia	(140, 141)
5	iron	9–29 μM for men, 7–27 μM for women^e^	185 μg/dL (33 μM)	iron tablets consumption case: death due to multiple organ failure after 48 h of ingestion (serum iron levels are best estimated within 2–6 h)	(142−144)
			360 μg/dL (64 μM)	serious toxicity: hypotension, metabolic acidosis, excessive irritability, severe dehydration: decreased skin turgor, sunken eyes, dry oral mucosa, and vomiting	
6	calcium	total calcium levels 2.2–2.6 mM and an ionized calcium level 1.15–1.33 mM	15.3 mg/dL (3.8 mM), 11.3 mg/dL (2.8 mM)	renal colic, confusion, lethargy, and weakness; reported passing small stones	(145−147)

a Nutrient concentrations are presented here for a comparative study. The specific values might differ depending on which reference is checked. Since these concentrations are reported as serum or plasma concentrations, there is no distinction between free and complex forms. Toxic serum concentrations are from case studies and are not meant as guidelines.

b Plasma concentration.

c Blood concentration.

d Copper: most of the copper in plasma is bound, with the majority being bound to ceruloplasmin and then a smaller portion to albumin, transcuprein, and small peptides or amino acids.^141^

e Iron: the plasma concentration includes complexed forms such as Hb and transferrin.^143^

**Table 3:** Therapeutic and Toxic Plasma Concentrations of Select Drugs^a^

#	drug	therapeutic plasma concentration range	toxic plasma concentrations (case studies)		ref
1	propranolol	0.0053–0.3 μg/mL (20 nM–1.15 μM)	10.2 μM, 13.5 μM	death	(165, 166, 175)
2	acetaminophen	5–20 μg/mL (33–132 μM)	960 μM	renal failure and then death	(21, 151, 152, 176−178)
3	warfarin	0.6–2.6 mg/L (1.9–8.4 μM),	INR 9.3	hemorrhage	(157−160, 179, 180)
		3.1–3.9 mg/L (10–12.6 μM), INR 2.0 to 3.0			
5	digoxin	0.5–2 ng/mL (640 pM – 2.5 nM)	42 ng/mL (54 nM)	death (ingested with suicide intentions along with propoxyphene hydrochloride)	(181, 182)
6	metoprolol	0.035–0.50 mg/L (130 nM – 1.8 μM)	4.7 mg/L (17.5 μM), 20 mg/L (75 μM)	death	(170−174)
7	fentanyl	2.51 μg/mL (7.5 nM)	13 μg/L (39 nM), 5 μg/L (14.8 nM), 48 μg/L (142 nM), 36 μg/L (107 nM)	death	(183, 184)

a Concentrations are presented here for a comparative study. The specific values might differ depending on which reference is checked. Toxic plasma concentrations are from case studies and are not meant as guidelines.

### 5.1 Comparison of CO’s Safety Margin with Some Commonly Encountered Biomolecules

For this section, we have selected a list of common biomolecules essential for the normal physiological functions of humans, including glucose, insulin, phenylalanine, and catecholamines (epinephrine). For example, glucose is an essential nutrient with a basal level of 3.9–5.7 mM (fasting) (Table 1, entry 1).^114^ However, slightly elevated levels at 5.7–6.4 mM are considered prediabetic (fasting), and >6.5 mM is diabetic (fasting) (Table 1, entry 1).^114^ At a concentration higher than 11 mM, glucose becomes potentially life-threatening.^115^ The safety margin is about twofold. In comparison, CO has a basal level of 1–2% COHb (about 75–150 μM) and does not show acute adverse effects at up to 6.3% in clinical trials or 9% in smokers. Further, CO at 2–4-fold of the basal level gives therapeutic effects in animal models. Such comparisons indicate a safety margin for CO comparable to or higher than that for glucose. Similarly, insulin is essential for regulating the glucose concentration and is a drug used to treat certain types of diabetes. However, it is also a choice of suicide drug among doctors.^116^ In one study, a patient of a suicide attempt with dangerous hypoglycemia had an insulin level of 402 μIU/mL, whereas the reference level is of 112 μIU/mL (112 μIU/mL at random sampling, <25 μIU/mL while fasting, and 18–276 μIU/mL at 1 h after glucose administration) for the normal population.^116−118^ This represents a difference of less than fourfold. Even with an essential amino acid, phenylalanine, the safety margin is only at 2–6-fold. Specifically, normal phenylalanine concentrations are in the range of 41–68 μM for adults and 26–86 μM for children (Table 1, entry 7).^119^ However, elevated levels at 121 μM due to deficiencies in metabolism lead to weakness in working memory and attention. At 364 μM, it is considered phenylketonuria (PKU) with serious impacts on cognitive functions and mental development.^120,121^ Such analysis shows a comparable or higher safety margin for CO compared to glucose, insulin, and phenylalanine. There are other similar cases in Table 1 that are not discussed in detail.

### 5.2 Comparison of CO’s Safety Margin with Inorganic Ions

Human bodies require inorganic ions within a narrow concentration range for normal functions. Table 2 summarizes the physiological and harmful levels of some metal ions and phosphate. As an example, potassium is essential for muscle functions, with a normal range of 3.6–5 mM.^128^ Seemingly minor deviations could have serious impacts on cardiac functions. Specifically, slightly elevated levels in the range of 5.5–6.5 mM lead to tall, peaked t-waves in EKG; levels in the range of 6.5–7.5 mM lead to the loss of p-waves; levels in the range of 7–8 mM lead to widening of the QRS complex; and levels in the range of 8–10 mM lead to cardiac arrhythmia, a sine wave pattern, or asystole.^129^ The safety margin with potassium is less than onefold and less than that of CO. Another example is magnesium, which is a vital mineral. Small deviations in magnesium concentration could have serious impacts on neuromuscular and cardiac functions. Specifically, the normal physiological range of magnesium is 0.75–0.95 mM.^130^ However, 2–4.5 mM magnesium can lead to disappearance of deep tendon reflexes, and 5 mM can lead to muscle weakness that proceeds to flaccid paralysis of voluntary and/or respiratory muscles, resulting in depressed respiration.^131^ Additionally, magnesium is also cardiotoxic, with findings of prolonged PR intervals, increased QRS duration, and QT intervals in EKG at 3 mM. At 7 mM, mild bradycardia and occasionally complete heart block as well as cardiac arrest can occur.^131^ The safety margin with magnesium is about seven fold, which is comparable to that of CO. Table 2 has other examples showing similar scenarios for many ions.

### 5.3 Comparison of CO’s Safety Margin with Some Commonly Used Drugs

Dose response is a central tenet of modern pharmacology and toxicology.^148^ The separation of therapeutic and toxic is in the dosage. This is true not only for CO but also for essentially all medications. In order to assess the safety of CO as a potential therapeutic agent, it is important to compare it against commonly used drugs. Table 3 summarizes therapeutic and toxic plasma concentrations of some common drugs.

As an example, the case of acetaminophen is discussed because it is generally considered as a very safe drug and has earned the status of “over the counter”. However, each year, there are about 56 000 patients seeking treatment for acetaminophen overdose or toxicity.^149^ In case studies, hepatotoxicity has been reported at a serum plasma concentration of 105 mg/L (695 μM, 2 h after ingestion), with an accidental dosage at 240 mg/kg in a 3 year old, which is fourfold higher than the allowable level (60 mg/kg).^150^ In adults, the therapeutic range of acetaminophen is 33–132 μM (Table 3, entry 2). In a case study, a 32-year-old woman had an acetaminophen overdose, which caused renal failure and then death 10 days after admission. The acetaminophen plasma concentration was found to be 960 μM, sevenfold higher than the peak therapeutic plasma concentration.^151,152^

Another example is warfarin. It is a commonly prescribed blood thinner used by 20 million patients each year.^153^ However, the small safety margin of this medication and significant individual variations are such that prescription levels need to be titrated for individual patients.^154^ According to the National Poison Data System, there were 1539 patients in 2021 and 1336 patients in 2022 treated in hospitals for warfarin ingestion.^155,156^ Because of the narrow safety margin of warfarin, maintaining the therapeutic concentration is difficult, and excessive blood thinning is commonly observed. The therapeutic range used in clinical practice is measured in a supratherapeutic international normalized ratio (INR). For normal patients who are not on any anticoagulant, the INR is usually 1 (it is a ratio, so no units).^157−159^ The therapeutic INR range is between 2.0 to 3.0 for patients who are undergoing anticoagulant therapy. INR levels above 4.9 are considered critical values and increase the risk of bleeding.^157−159^ In a case study, INR 9.3 resulted in hemorrhage due to warfarin toxicity.^160^ These levels all need to be assessed for individual patients. Further highlighting its safety issue is the fact that warfarin is also used as rat poison because of its effectiveness in extinguishing rats after ingestion.^161^

Another example is propranolol, which is used to treat various conditions, including cardiovascular conditions, psychiatric conditions, and PTSD.^162,163^ Yearly prescriptions are in millions in the United States.^164^ Such a widely prescribed drug also has a narrow safety margin. Therapeutic effects are observed at a plasma concentration of 20 nM to 1.15 μM (Table 3, entry 1).^165^ In one case report, a plasma concentration of 10.2 μM (3 h after admission) led to death (Table 3, entry 1) as a result of an suicide attempt.^166^ Such an example clearly shows the narrow safety margin of this widely prescribed drug. Adverse effects and death can be seen even at doses that are onefold and ninefold higher than the therapeutic plasma concentration, respectively (Table 3, entry 1).

Another example is metoprolol, an antiarrhythmic drug and a class II selective beta blocker.^167^ Metoprolol is used alone or in combination with other drugs to treat high blood pressure.^168^ It is used for chest pain due to poor blood flow and a number of conditions involving an abnormally fast heart rate.^168^ In 2021, metoprolol was prescribed around 65.5 million times in the United States. Such a widely prescribed drug also has a narrow safety margin.^169^ Therapeutic effects are observed at plasma concentrations of 0.035–0.50 mg/L (130 nM – 1.8 μM) **(**Table 3, entry 6), and toxic effects are observed at plasma concentrations higher than 1 mg/L (3.7 μM).^170−174^ In one case study of ingesting metoprolol with suicidal intentions, the blood concentration was found to be 4.7 mg/L (17.5 μM).^172^ In another case of ingestion of metoprolol with suicidal intentions, the blood concentration was found to be 20 mg/L (75 μM), along with a blood alcohol concentration of 0.25 g/100 mL (54 mM).^173^Table 3 has other examples showing similar scenarios for many drugs.

All these examples indicate that the safety margin of CO is comparable to those of many commonly used drugs.

## 6 Delivery Routes Make a Difference in CO Toxicity and COHb Should Not Be Regarded as the Single Parameter to Predict Toxicity

One of the most challenging issues in studying CO safety and efficacy in a dose-dependent manner is the lack of a reliable indicator or biomarker of pharmacologically and toxicologically relevant concentration. Because of the low water solubility of CO (∼1 mM at 1 atm), the concentration of free CO in the blood is not a reliable number that can be readily obtained. Therefore, COHb is the most convenient and thus commonly used term in describing the CO exposure level. However, there have been many studies that indicate the unreliable nature of relying only on the COHb level to correlate with clinical observations. Likely, a combination of several factors is responsible for CO toxicity, including the rate of CO intake relative to the binding kinetics with hemoglobin and other hemoproteins, tissue concentrations, the binding mode of CO with hemoglobin, and total CO intake. However, a common perception is that CO poisoning involves the binding of carbon monoxide to hemoglobin in red blood cells. As an example of how widespread this perception is, we asked Chat Generative Pretrained Transformer (ChatGPT) about the mechanism of CO poisoning; it gave an answer stating that the mechanism of CO poisoning involves the binding of carbon monoxide to hemoglobin in red blood cells.^185^ Such an answer reflects a widely held belief that an elevated level of COHb is the cause of death of CO poisoning *per se* because this leads to the diminished ability for hemoglobin to carry oxygen. Emerging evidence indicates that at minimum this is an oversimplification.^18^ There is experimental evidence to show that the lethality of CO at the same COHb level is different depending on delivery routes, with inhalation being most lethal.^33^ There is clinical evidence to show a mismatch of the COHb level and clinical presentations in terms of symptoms or death. There are likely different explanations for animal model work and clinical observations, which are discussed later in this section. Nevertheless, the evidence is clear that COHb is not the single parameter of the CO exposure level that can be correlated with toxicity. Further, the delivery route and likely the composition of the four COHb forms make a fundamental difference in CO toxicity. Below we provide detailed analyses of these issues themselves and in the context of examining the issues of safety margins and therapeutic applications of CO.

As discussed earlier, endogenously produced CO is largely bound to hemoglobin and eliminated through exhalation, with a small percentage of oxidation to CO_2_ by cytochrome *c* oxidase. About 10–15% of CO is bound to other hemoproteins located in extravascular tissues.^186^ Less than 1% is dissolved in bodily fluid.^187−189^ In terms of its chemical reactivity, CO is different from the other two endogenously produced gaseous molecules, NO and H_2_S. CO is chemically inert in the body in the absence of enzyme catalysis. The stability of CO arises from its triple bond, which is the strongest chemical bond known. In terms of noncovalent interactions, CO is too small to offer the necessary binding energy to have meaningful affinity for biomolecules such as enzymes and receptors to be biologically significant unless there is a metal involved. All known biological functions of CO occur through binding to metal ions, primarily hemoproteins in the ferrous form. The extraordinarily strong affinity of CO for metal atoms or ions in a low oxidation state is attributed to backbonding of d-orbital electrons of the metal into the π-antibonding orbital of C≡O. The CO adducts with hemoproteins are generally more stable and less reactive than the O_2_ adduct due to the π-backbonding from iron(II).^190,191^ Thus, hemoproteins generally have a higher binding affinity for CO than O_2_. As an example, the ratio of hemoglobin’s affinity for CO averages about 240-fold that of O_2_,^60^ with factors such as pH, O_2_ level, and the presence of 2,3-DPG being important in determining the specific affinity ratio.^19,192^ Hemolysis causes the release of hemoglobin from red blood cells (RBCs), which becomes a highly toxic substance in plasma.^193^ Cell-free oxy-Hb can be easily oxidized to cell-free met-Hb by oxidants such as H_2_O_2_ and NO. Endogenously produced CO binds to ferrous hemoglobin, leading to an oxidation-resistant CO–Hb complex. This antioxidant effect inhibits the dissociation of cell-free hemoglobin and thus controls the release of heme from hemoglobin.^194−196^ Cell-free COHb is more stable than cell-free oxy-Hb, thus reducing the toxicity of cell-free hemoglobin.^195,196^ In experiments for determining the effect of azide on the autoxidation of hemoglobin, an increase in the reaction rate constant from 1.1 × 10^–3^ to 18.1 × 10^–3^ min^–1^ was observed when the O_2_Hb/COHb ratio increased from 0.02% to 100% O_2_Hb. The initial rate of autoxidation was also observed to significantly increase from 0.011% min^–1^ in 100% COHb to 3.7% min^–1^ in 100% deoxy-Hb.^197^ Intraperitoneal administration of the CO scavenger hemoCD to mice leads to a “CO depleted” state, allowing the study of various effects of CO.^198^ OxyHb is easily oxidized to MetHb, leading to heme release, which triggers HMOX-1 expression.^195,196^ Based on in vitro experiments reported by Hebbel and colleagues, the apparent rate constants of heme transfer from hemoglobin to hemopexin (Hpx) of oxy-HbA, oxy-HbS, COHb, and MetHb were measured as 0.014, 0.024, 0, and 0.923 h^–1^, respectively.^195^ All these point to the contribution of COHb formation to slowing down the oxidation of hemoglobin, heme release from MetHb, and thus reduction in the toxic effects of cell-free hemoglobin.^198^ As such, the formation of COHb at a certain level not only is not the cause of lethal effects per se but also offers protection against Hb-induced toxicity under certain conditions that lead to massive hemoglobin release, such as organ injury and inflammation.

Another piece of evidence suggesting the protective roles of COHb formation comes from elephant seals. It is important to note that elephant seals go through deep-diving cycles as part of their daily routine. Such deep dives can last 20 min with arterial hemoglobin O_2_ saturation below 80%.^199^ As a result, elephant seals experience routine hypoxia and reoxygenation repeatedly. Given the known harmful effects of the ischemia–reperfusion process in humans,^200^ it is intriguing to think of the kind of mechanism(s) that elephant seals use to attenuate the damaging effects of this frequent hypoxia–reoxygenation cycle. Interestingly, the average COHb level was found as 8.7% in northern elephant seals.^201^ The maximum COHb level was found to be 10.4% in an adult elephant seal. This is comparable to the COHb level (14%) set by the U.S. FDA in human clinical trials for a kidney transplant study.^32^ Because of the organ-protective effects of low-dose CO, one wonders whether northern elephant seals evolved to use the high concentration of endogenous CO and COHb to offer cyto- or organ-protective functions during repeated ischemia–reperfusion.^201^

CO has a high tendency to replace O_2_ and further stabilizes the relaxed state (R-state) of hemoglobin, resulting in a shift of the oxyhemoglobin dissociation curve to the left and reducing both the oxygen-carrying capacity of hemoglobin and the O_2_ delivery tendency to tissues by hemoglobin.^187,202−206^ This is an important point for later discussions as well. Therefore, several studies have concluded that elevated COHb levels decrease the oxygen-carrying capacity of blood and produce hypoxia in tissues, which is traditionally considered as the primary cause of death in CO poisoning.^207−213^ Based on early studies of CO poisoning, it is reasonable that the blood COHb percentage (COHb%) is most frequently used as a biomarker for CO poisoning.^214,215^ Although an elevated COHb level is an important benchmark for assessing exposure to exogenous CO, it is widely recognized that there is a mismatch between clinical outcomes and COHb levels.^216−221^ However, it is also important to note that clinical studies often are based on patients who have experienced CO poisoning from fires or car exhausts. Both involve many other components such as cyanide and various oxidized nitrogen (NO_*x*_) species, which could have contributed to clinical symptoms and complications in result interpretation.^187,206,222−224^ Additionally, the elimination half-life of COHb is highly variable and depends on cardiopulmonary function, leading to an unreliable representation of the true extent of CO poisoning by COHb.^187,225^ Thus, even when the COHb level is in the normal range at the time of diagnosis, patients can develop severe neurologic injuries and die of “CO poisoning” in some cases.^226−228^ Furthermore, neurologic injury and CO cardiotoxicity that often develop in “CO poisoning” also cannot be explained only through the hypoxemic hypoxia caused by the elevated COHb level.^221,229−233^ Therefore, there is a growing consensus that elevated COHb levels are not always a direct and/or primary cause of acute toxicity. In the end, it should be emphasized that interpreting postperspective clinical data in humans requires considering many more factors, including significant variations in the health states of individuals and differences in sources of CO. Therefore, other factors such as cyanide production in fire, the presence of a high concentration of NO_*x*_ species,^223,224,228^ and severe underlying health problems in individuals all complicate result interpretation and the correlation with COHb.

**Figure 5:**
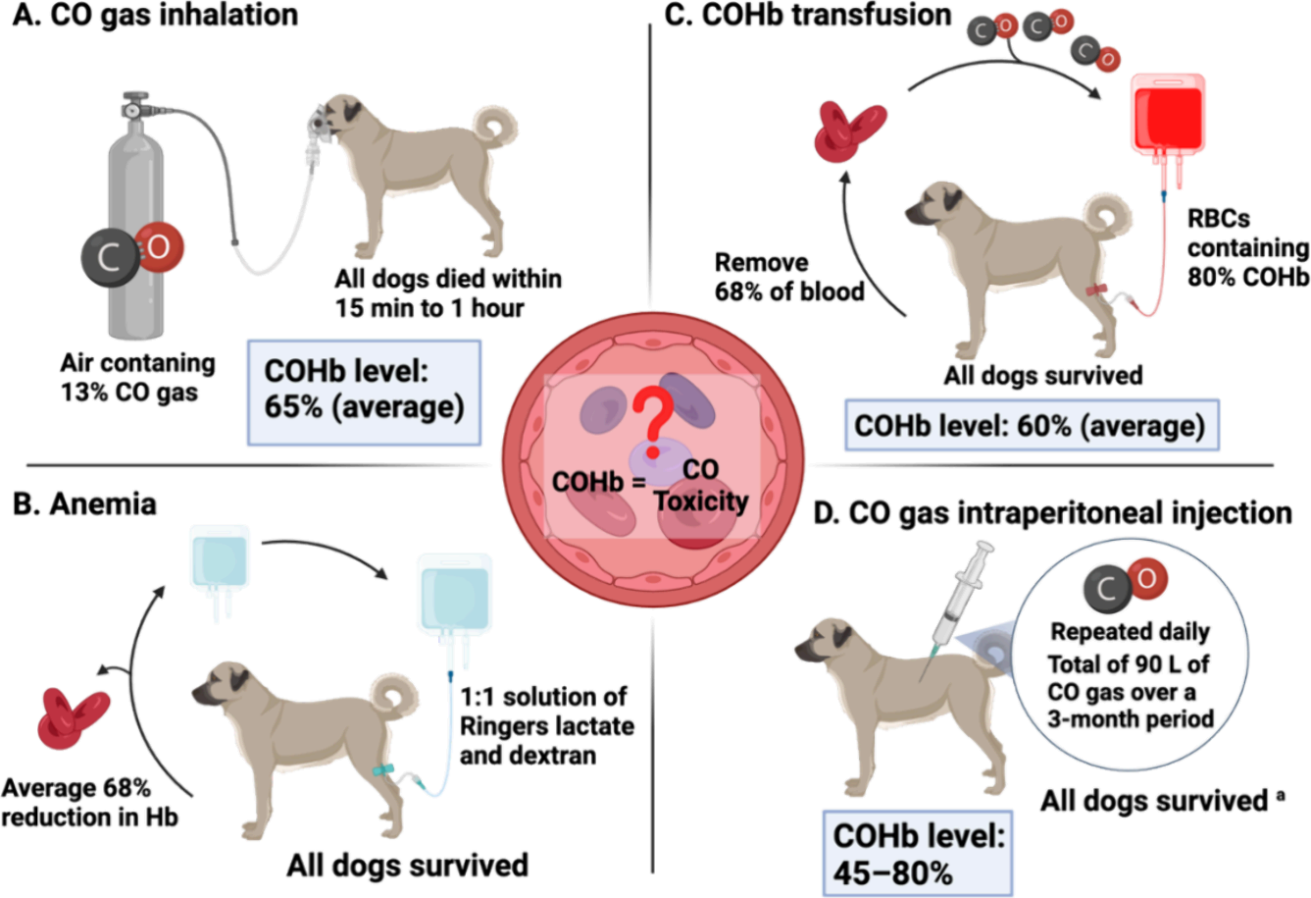
Mechanisms of the CO toxicity. ^*a*^Three of the 10 dogs died on days 15–42 from unrelated causes.^234^ Figure adapted from images created with BioRender.com.

The mechanisms of CO toxicity were discussed as early as 1975.^234^ Leo Goldbaum conducted a series of experiments using dogs to examine this issue.^34,234−236^ Specifically, there were four types of experiments. First, dogs exposed to air containing 13% CO gas died within the window of 15 min to 1 h, with the average COHb level being 65% at the time of death (Figure 5A).^234^ On the other hand, when dogs were bled and placed in an anemic state with an average of 68% reduction in hemoglobin content and then transfused with a 1:1 solution of Ringers lactate and dextran, all the dogs survived indefinitely (Figure 5B).^234^ In a third type of experiment, 68% of the blood was removed and replaced with red blood cells (RBCs) containing 80% COHb, leading to a COHb level of 60%. Though the dogs achieved a similar level of COHb that was lethal when achieved through inhalation, all the dogs survived (Figure 5C). In a fourth type of experiment, maximal COHb levels of 45–80% were achieved by i.p. injection of CO gas, with injections repeated daily for a total of 90 L of carbon monoxide over a 3-month period (Figure 5D). Three of the 10 dogs died on days 15–42 from unrelated causes.^34,234−236^ These experiments clearly demonstrate that COHb per se does not explain the cause of CO poisoning even at high levels, and the apparent low oxygen saturation level (i.e., low O_2_Hb percentage) alone also does not seem to be the cause of CO poisoning. Further, the route of CO administration makes a fundamental difference in its lethal effects. Only inhalation of gaseous CO showed lethal toxicity in these experiments. Below, we provide our analysis of the reasons for the observed differential toxicity depending on the route of administration from two aspects: free CO content in the blood to rapidly access vital organs and COHb compositions.

Following inhalation, inhaled CO binds to hemoglobin in the R-state during the gas exchange in the lungs due to the 180-fold higher affinity of hemoglobin for CO than for O_2_.^237^ However, the equilibration time of CO binding to hemoglobin in red blood cells (RBCs) is on the scale of minutes based on the kinetic and thermodynamic data of hemoglobin in the R-state.^33^ Goldbaum’s experiment also support that the binding of CO to RBCs is not rapid. In his experiments, it was found that only 26% of hemoglobin was converted to COHb in 5 min, and it took 20 min to reach almost full saturation when blood was shaken in a 100% CO atmosphere in vitro.^34^ It should be noted that an “average” person pumps 5–6 L of blood per min, which is approximately the equivalent of total blood volume. Therefore, it is reasonable to assume that inhaled CO is not captured by hemoglobin soon enough in the lungs, travels in plasma, and reaches vital organs rapidly with the flow of arterial blood. As discussed above, it has been found that CO can bind to hemoproteins in tissues, including myoglobin (Mb), cytochrome *c* oxidase (C*c*O), and cytochrome P450, as summarized in a recent review.^19^ There have been efforts to examine tissue distribution after CO inhalation in animal models.^238,239^ CO concentrations are reported to increase in all tissues after CO inhalation.^238,239^ In one set of experiments, rats were allowed to inhale 400 ppm of CO gas for 20 min. CO concentrations in tissues were observed to increase quickly in the first 5 min of exposure and then plateaued off near the saturation capacity of the tissues after 10 min of CO exposure, even though the blood COHb level linearly increased afterward.^239^ This was proposed to mean that COHb is formed to prevent the accumulation of excess CO in tissues and thus to offer protection. The implication is also that COHb is not the lone cause of CO toxicity per se.^239^ CO can inhibit mitochondrial respiration by binding to ferrous heme *a*_3_ in the electron transport chain of C*c*O, which might be the most important mechanism of CO’s toxicity at the molecular level.^206,237,240−242^ CO’s effect on the activity of C*c*O is dependent on the O_2_ concentration. Specifically, tissue hypoxia may lead the electron transport chain to be in a more reduced state, which is more favorable for CO binding.^243^ Once CO binds to heme *a*_3_, O_2_ utilization is reduced in mitochondria, leading to decreased ATP production, increased production of ROS, and finally irreversible damages to tissues.^206,244,245^ In human acute CO poisoning, Miró and colleagues studied complex II, complex III, and C*c*O (complex IV) in the mitochondria of lymphocytes from three patients with acute CO poisoning.^246^ The activity of C*c*O was 24% of the normal level, with the average COHb level at around 17%. Three days later, the COHb level had decreased to about 2.1%; however, the activity of C*c*O was still only at 60% of the normal level. After 12 days, the activities of C*c*O recovered, and the average of the COHb level was 1.9%. Additionally, the activities of complex II and complex III did not significantly change compared to normal levels during this study. In mouse experiments for acute CO poisoning, mice were first exposed to 3% CO gas for 4.5 min. After CO exposure, hearts were removed immediately upon death or after 20 min of air ventilation to measure the inhibition of cardiac respiration by CO. The respiration of the hearts from CO-treated mice was significantly inhibited to a respiration rate of 58 ± 19% of the controls. The activities of C*c*O, complex I, and complex II were also significantly decreased after the CO exposure. Further experiments in mice at hypoxic conditions (2% oxygen) with and without the presence of CO-saturated buffer led to the conclusion that only the activity of complex IV was significantly inhibited, with the CO-exposed activity level at 33 ± 5% of the controls. Such results led to the conclusion that CO-induced inhibition of mitochondrial respiration is due to the inhibition of C*c*O.^237^ CO was also found to bind C*c*O in the brain through a series of experiments with rats.^241,242^ Overall, even though there are also other proposed mechanisms of CO toxicity, CO’s inhibition of C*c*O is considered a very important mechanism. Inhaled CO allows for the rapid engagement of C*c*O in vital organs, which may significantly contribute to CO toxicity.

**Figure 6:**
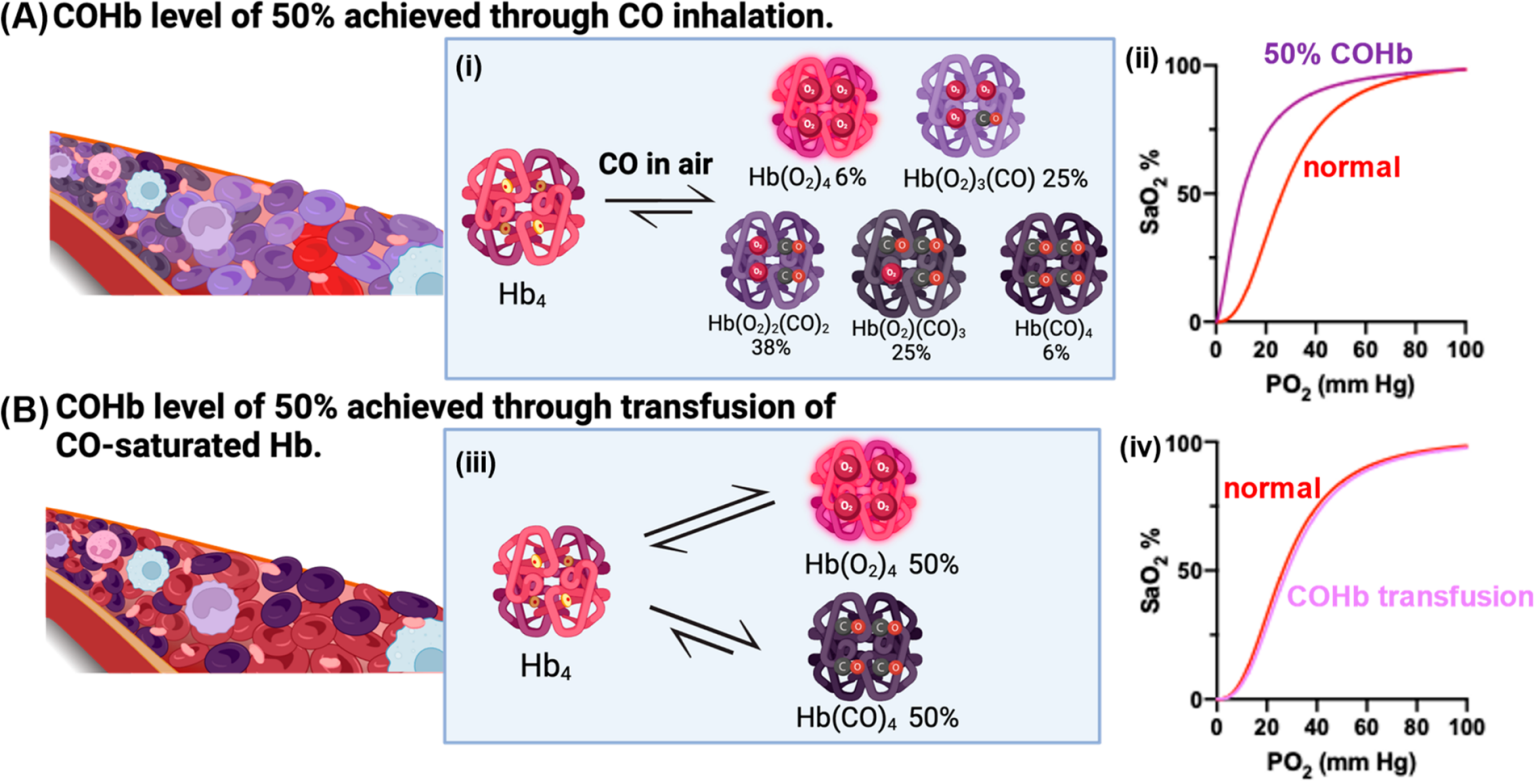
Differences in Hb functions between CO gas inhalation and COHb transfusion. (A) CO gas inhalation leading to a COHb level of 50%: (i) Calculated distribution of all forms of Hb in the blood when CO gas binds to Hb.^247,248^ (ii) COHb shifts the oxygen–hemoglobin dissociation curve to the left and transforms it into a hyperbolic shape. The percent saturation of Hb with oxygen (SaO_2_%) is plotted against the partial pressure of oxygen (PO_2_).^187^ (B) Transfusion of CO-saturated Hb (i) leads to two distinct forms of Hb in the blood and (ii) allows the oxygen-hemoglobin dissociation curve to remain the same as the normal curve when SaO_2_% is plotted against PO_2_.^249^ Figure adapted from images created with BioRender.com.

With regard to why the route of delivery makes a difference in terms of CO toxicity, we would like to analyze this in detail here because it is a very important topic in the context of using CO as a therapeutic agent. CO binding to hemoglobin in cases of CO poisoning through inhalation shifts the oxyhemoglobin dissociation curve to the left, and the sigmoid curve becomes more hyperbolic due to increased cooperative binding of O_2_ (Figure 6A(i)). This shift makes it more difficult for hemoglobin to release O_2_ to tissues even in comparison to the same degree of hemoglobin reduction caused by anemia (Figure 6A(ii)). This is because of the lowered oxygen partial pressure (*P*^O2^) needed to release oxygen. For example, the hemoglobin affinity for oxygen as measured in *P*_1/2_^O2^ (*P*^O2^ at 50% O_2_Hb) in a CO poisoning patient with 50% COHb is 16 mmHg. This means 50% oxygen release at 16 mmHg. On the other hand, *P*_1/2_^O2^ in patients with acute anemia (50% reduction of Hb concentration) is 26 mmHg, which is similar to that in a healthy person (*P*_1/2_^O2^ = 26.9 mmHg at pH 7.4 and 37 °C).^69,250^ This need for a much lower *P*^O2^ for CO-bound hemoglobin to release oxygen creates severe tissue hypoxia.

The difference in oxygen-delivery ability between a 50% reduction in hemoglobin content (anemia) and a 50% COHb content also helps explain the difference in the ease of CO poisoning depending on the delivery route. In essence, 50% COHb when achieved via inhalation means much more than a 50% reduction in available hemoglobin for oxygen delivery. For an in-depth explanation, we need to look at the technical details, and the matter comes to the “occupancy rate” of hemoglobin by CO.

**Figure 7:**
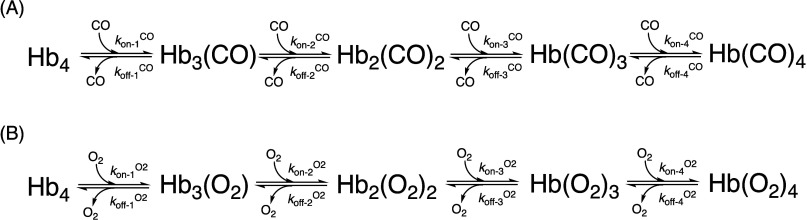
Binding equilibria between hemoglobin and (A) CO and (B) O_2_.

First, adult human hemoglobin is a tetrameric protein of two pairs of identical peptide chains, Hb_α_ and Hb_β._ Binding of the first oxygen increases the affinity of the remaining binding site for subsequent loading through allosteric effects.^65^ Oxygen off-loading follows a similar principle except in the reverse fashion.^251^ The binding of CO to hemoglobin is similar to that of oxygen, except the affinity is higher. For CO binding to hemoglobin, there are several permutations of the final form (mixture of Hb_4_(O_2_)_4_, Hb_4_(O_2_)_3_(CO), Hb_4_(O_2_)_2_(CO)_2_, Hb_4_(O_2_)(CO)_3_, and Hb_4_(CO)_4_).^252−255^ Before we discuss the possible compositions of the four forms of COHb, it is important to bring in the issue of association constants *K*_a_ for each step of the binding of CO and O_2_ to hemoglobin (Figure 7). There have been extensive efforts to study both the kinetic and thermodynamic parameters for each of the binding processes. There are some variations in the specific numbers obtained as well as the conditions used. Further, some of the numbers are calculated based on the on–off rates from different publications. Nevertheless, these numbers all present a very similar picture, as shown in Table 4. We focus our discussion on one set of data, which was based on a single set of experiments with CO. As one can see, the association constants (*K*_a_) are different for all four steps, with the second and fourth having higher affinity for CO and the first and third having lower affinity. There are a few observations to make with regard to these numbers. First, because each step has a different *K*_a_ for CO, the ratios among the four forms are likely to deviate from statistical distributions (next section). Second, the binding constants of CO in Table 4 are determined in the absence of O_2_ using CO gas in the presence of sodium dithionite. The mixed forms with both CO and O_2_ present are likely to be more complex and to be somewhat different from the binding with pure CO. Third, because hemoglobin has higher affinity for the second and fourth CO than the first and third CO by 22–80-fold, the composition likely favors paired forms, i.e., either Hb_4_(O_2_)_2_(CO)_2_ or Hb_4_(CO)_4._ As a matter of fact, hemoglobin’s affinity for the first and third CO is only 4–13-fold higher than that for the second and fourth O_2_ (Table 4). This means that the major difference in affinity between CO and O_2_ is observed in steps 2 and 4. Finally, even with this seemingly complex analysis of the binding affinity for hemoglobin toward CO, the real-life scenario in the presence of both O_2_ and CO is far more complex. There has only been limited experimental work on the binding affinity of hemoglobin for CO in the presence of oxygen or vice versa.^256,257^ Therefore, in analyzing hemoglobin binding with CO in the presence of O_2_, one has to be mindful of all of these considerations.

**Table 4:** Kinetic and Thermodynamic Parameters of CO and O_2_ Binding with Hemoglobin

	L = CO			L = O_2_		
step	*k*_on_^CO^ (M^–1^ s^–1^)^a^	*k*_off_^CO^ (M^–1^ s^–1^)^b^	*K*_a_^CO^ (M^–1^)^b^^,^^c^	*k*_on_^O2^ (M^–1^ s^–1^)^d^	*k*_off_^O2^ (s^–1^)^d^	*K*_a_^O2^ (M^–1^)^d^
Hb_4_ + L ⇌ Hb_3_L	2.52 × 10^5^	0.09	2.8 × 10^6^	1.77 × 10^7^	1.9 × 10^3^	9.3 × 10^3^
Hb_3_L + L ⇌ Hb_2_L_2_	1.76 × 10^6^	0.028	6.3 × 10^7^	3.32 × 10^7^	1.58 × 10^2^	2.13 × 10^5^
Hb_2_L_2_ + L ⇌ HbL_3_	2.43 × 10^5^	0.09	2.7 × 10^6^	4.89 × 10^6^	5.39 × 10^2^	9.07 × 10^3^
HbL_3_ + L ⇌ HbL_4_	7.2 × 10^6^	0.036	2.0 × 10^8^	3.3 × 10^7^	5.0 × 10	6.6 × 10^5^
	7.0 × 10^6^^e^	0.031^e^	2.2 × 10^8^^e^			

a Calculated from *K*_a_^CO^ and *k*_off_^CO^.

b Ref (247).

c Refs (247, 258, 259).

d Ref (260).

e Ref (261).

With all the discussions of the molecular events of the binding process between hemoglobin and CO, we now turn back to the different scenarios when presented with an experimentally measured COHb level of 50%. In one scenario, 50% COHb could mean that 100% of the hemoglobin has at least one CO bound to Hb (Figure 6A). Based on the calculation by Gibson and colleagues, the statistical distribution of all forms in blood with 50% COHb corresponds to 6% Hb_4_(O_2_)_4_, 25% Hb_4_(O_2_)_3_(CO), 38% Hb_4_(O_2_)_2_(CO)_2_, 25% Hb_4_(O_2_)(CO)_3_, and 6% Hb_4_(CO)_4_ (Figure 6A(i)).^247,248^ Adding all these together comes to 94% of all hemoglobin having at least one molecule of CO. This is a high “occupancy rate” and affects the delivery of O_2_ by nearly all of the hemoglobin molecules (or 94%). Such numbers are, of course, based on statistical distribution without considering the changing affinities of the four hemoglobin monomers in the process of successive CO binding (Figure 7). Nevertheless, the calculation presents a conceptually correct scenario of the distributed binding modes between CO and hemoglobin, which is not homogeneous. To the other extreme, experimentally determined 50% COHb could mean 50% of the blood hemoglobin is fully occupied (four CO per Hb) by CO and 50% of the hemoglobin is completely free of CO (Figure 6B). Not considering the effect of further scrambling, the second scenario is almost the equivalent of an anemic person with 50% of the normal hemoglobin content. It should be noted that anemia represents a decreased amount of hemoglobin to carry oxygen, but the oxyhemoglobin dissociation curve remains essentially the same (Figure 6B(ii)). Further, anemia in dogs given full-CO-saturated hemoglobin is a transient event. The half-life for clearing COHb in the blood is about 2 h for dogs with a high level of COHb.^236,262,263^ For example, when anesthetized dogs were allowed to inhale CO for 3 min, their COHb levels reached 20–43%. Such a high COHb level in arterial blood decreased exponentially within the first 15 min. This was followed by a slower linear phase over approximately 75 min. The half-life for clearance in dogs was measured as about 2 h for an initial COHb level of 20–43%.^262,263^ This means that CO in the form of transfused COHb can be eliminated through exhalation very quickly due to its short half-life. Therefore, there is enough CO-free hemoglobin to carry and deliver oxygen after transfusion of blood with COHb. This further means there is no attenuated release of the oxygen carried to the tissue (Figure 6B(ii)). Of course, this is with the assumption of little scrambling among the four possible forms of COHb and (O_2_)_4_Hb. This seems to be a safe assumption if one looks at the dog experiments described in Figure 5C. Now, we look at the first scenario, or 94% hemoglobin carrying at least one CO molecule, as shown in Figure 6A(i). This scenario would have both decreased oxygen-carrying capacity and a depressed ability to release/deliver whatever amount of oxygen carried with the hemoglobin to the tissue site (Figure 6A(ii)). Therefore, the first scenario described in Figure 6A represents a much higher risk in terms of the creation of dangerous levels of hypoxia. In the dog experiments described at the beginning of this section, administering 80% CO-saturated hemoglobin to achieve 65% COHb (Figure 5C) is more like the second scenario, with symptoms similar to anemia (Figure 5B and Figure 6B). On the other hand, breathing in CO and achieving 65% COHb almost certainly mean that nearly all the hemoglobin molecules carry at least one CO molecule as described in Figure 6a, leading to a much decreased ability to deliver oxygen to tissues and severe hypoxemic hypoxia. These analyses explain why the route of administration makes a difference in CO toxicity. However, we should note that the lack of lethal effects when a high COHb level was achieved through noninhalation delivery should NOT be interpreted to mean the lack of undesirable effects. Severe anemia is known to have serious health consequences. A 50–80% reduction of available hemoglobin for O_2_ delivery is expected to have serious undesirable, if not lethal, effects in humans.

In another set of experiments by Drabkin et al. in the 1940s to understand the effects of CO in preventing the dissociation of oxyhemoglobin,^264−266^ dogs were exposed to an atmosphere containing CO until the blood hemoglobin reached 75% saturation with CO. These animals collapsed with evidence of cardiac and respiratory failure. In those that survived, extensive necrotic changes were later found in the brain and heart. In another group of dogs, the CO concentration in the blood was brought to 75% saturation by partial replacement transfusion of washed RBCs that were completely saturated with CO. No signs characteristic of anoxia were observed, and no myocardial or cerebral necrosis were observed. In addition, the rate of CO elimination was described to be twice as rapid as that from dogs that had inhaled the gas. Based on the dissociation curve of oxy-Hb in the non-CO-exposed group and in the group with 75% COHb, the dogs that had inhaled CO actually do not have 25% of the normal amount of oxygen available to their tissues but instead only 11%. On the other hand, the dogs that were transfused with CO-saturated erythrocytes had available to their tissues 25% of the amount of oxygen normally carried by the blood in the absence of CO. Such results are consistent with the analysis in the preceding paragraph. It should be noted that the dissociation constant *K*_d_^CO^ was measured to be 3.6 × 10^–7^ and 5.0 × 10^–9^ M (*K*_a_^CO^ = 2.8 × 10^6^ and 2.0 × 10^8^ M^–1^ respectively, Table 4) for the first (Hb_4_(CO)) and last CO (Hb_4_(CO)_4_), respectively.^247,260^ This means that the CO from Hb_4_(CO)_4_ is not nearly as readily available to engage other targets as the CO from Hb_4_(CO) or Hb_4_(CO)_3_. Such *K*_d_ differences also help explain the seeming lack of scrambling among the different forms of COHb and hemoglobin. Much more work is needed to understand the implications of the thermodynamics and kinetics of CO binding (and dissociation) in different forms of COHb and Hb.

When CO gas is injected into the GI or intraperitoneal cavity, it is exposed to peripheral tissues where the pH is low, CO_2_ contents are high, and 2,3-DPG is probably present. All these factors mean the existence of hemoglobin in the T-state, with *K*_d_ being 1.1 μM for CO^267^ and 420 μM for O_2,_^267,268^ leading to a 390-fold higher affinity for CO. Additionally, the kinetic association constant (*k*_on_) of the first CO to bind to Hb_4_ is 2.52 × 10^5^ M^–1^ s^–1^, and the *k*_on_ of the last CO to bind to Hb_4_(CO)_3_ is 7.2 × 10^6^ M^–1^ s^–1^. The dissociation constant *K*_d_^CO^ was measured to be 3.6 × 10^–7^ and 5.0 × 10^–9^ M (*K*_a_^CO^ = 2.8 × 10^6^ and 2.0 × 10^8^ M^–1^ respectively, Table 4) for the first and last CO, respectively.^247,269^ Further, with the high CO concentration at the delivery site (e.g., intraperitoneal injection of CO gas) and low O_2_ partial pressure (typically 5% in peripheral tissues),^270^ the scenario heavily favors CO binding, likely leading to hemoglobin being mostly fully loaded with CO (i.e., four CO molecules/Hb) as in the case of Figure 6B. Such analyses, coupled with slow CO binding in the lungs as discussed earlier, are consistent with inhalation being the most lethal form of CO delivery and favor delivery via a noninhalation route with an enhanced safety margin.

Altogether, numerous experimental results have proven that the COHb level does not reliably correlate with CO toxicity, regardless of whether it is formed under normal conditions or under the conditions of CO poisoning. Route of delivery makes a fundamental difference in terms of CO toxicity. At the same level of COHb, inhaled CO seems to be the most life-threatening. If techniques are available to deconvolute the COHb occupancy distribution (i.e., percentage of hemoglobin that has one, two, three, or four CO molecules), it will help further clarify the technical details why delivery form makes a difference.

With the discussions of CO delivery forms alternative to inhalation, it is important to note that many forms of CO donors have been developed, including metal-based CO-releasing molecules (CORMs)^271,272^ and organic CO donors that are photosensitive, ROS-sensitive, ultrasound, mechanical force-sensitive, or chemoexcitation-sensitive.^273−280^ There have also been efforts to develop metal-based CORMs for triggered CO release,^281−285^ trapped CO,^286^ and CO solution.^6,286^ In 2014, we reported the first organic CO prodrugs by taking advantage of a cheletropic reaction for CO release from a norbornadienone scaffold.^287^ This was followed by a series of reports of organic CO prodrugs of various properties, including one that uses saccharine and acesulfame as carrier molecules for CO delivery, and immobilized CO prodrugs.^288^ Among the large number of CO donors published, CORM-2, CORM-3, CORM-401, and CORM-A1 are probably the most well-known.^272^ Due to their commercial availability and ease of use, these four CORMs have been widely used as CO surrogates in a large number of studies examining the biological effects of CO.^272,289^ However, recent years have found various issues with these CORMs,^271,290^ raising caution in developing CO donors for noninhalation delivery forms. Careful attention is needed to understand the CO release properties/stoichiometry, conditions that affect CO release, CO-independent activity, and sometimes the chemical reactivity of a donor molecule before attributing observed biological activity to the “CO released”. Though this is a different topic, it is a critical issue to consider in this field in developing alternative CO delivery forms.^19,50^

With the enhanced safety margin for noninhalation delivery forms, there is still room for improvement by using targeted delivery. This is discussed in the following section.

### 6.1 Targeted Delivery

It is conceivable that targeted delivery of CO can increase the CO safety margin even further when compared to traditional CO donor forms. This does not require much explanation. Localized delivery is one way to achieve this. Photosensitive,^273,291^ mechanic-force-sensitive,^278,292^ chemoexcitation-sensitive,^277^ and peroxynitrite-sensitive^279^ donors all offer this type of possibility. Local delivery such as a foam formulation,^286^ a biodegradable gel,^293^ and coated tablets^285^ also offers the chance for selective delivery and is expected to improve the safety margin in a systemic sense. Targeted delivery based on events at the molecular level or at the organelle level may offer an even further enhanced safety margin. Along this line, there has been ROS-sensitive CO delivery.^54,55,294^ This is predicated on the idea that inflammation and organ injury tend to lead to elevated levels of ROS. Another approach is organelle-targeting. For example, there have been reports of mitochondrion-targeted CO delivery^53,295^ because of the mitochondrion’s central role in the proposed mechanism of action(s) by CO.^19^ We reported the first example of mitochondrion-targeted CO delivery with improved potency.^53^

**Figure 8:**
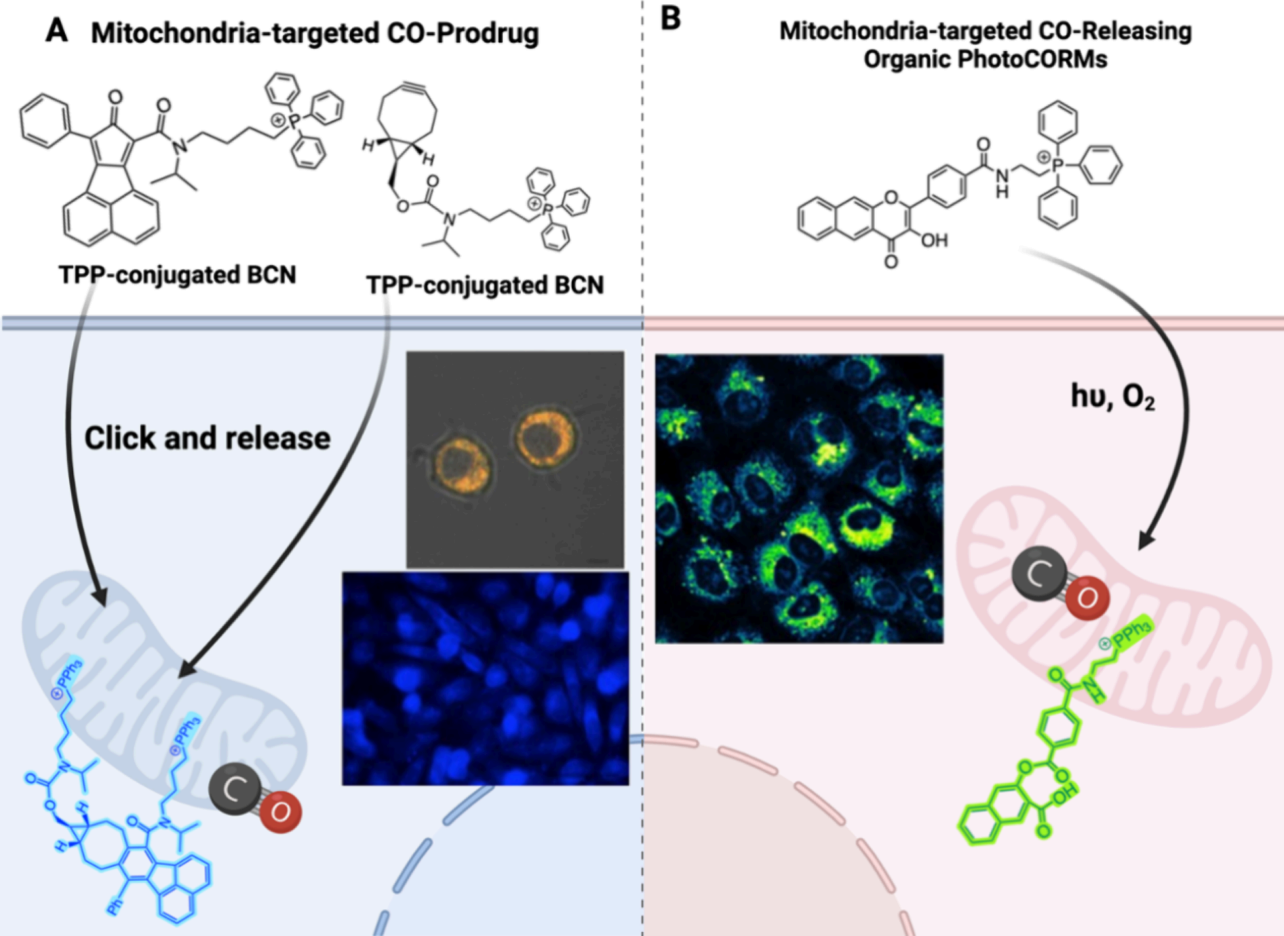
Mitochondrial-targeted CO delivery. (A) An enrichment-triggered release approach to target CO to the mitochondrion. Fluorescence images show colocalization of the product following a Diels–Alder click reaction with mitochondrial tracker MitoTracker Deep Red in RAW264.7 cells. Adapted from ref (53). Copyright 2018 Springer Nature. (B) Mitochondria-targeted PhotoCORM approach for mitochondrial bioenergetics studies. Confocal microscopy image showing colocalization of mitochondrion-targeted PhotoCORM with MitoTracker Red CMXRos in A549 cells. Adapted from ref (295). Copyright 2018 American Chemical Society. Figure adapted from images created with BioRender.com.

Because this Perspective is not focused on CO delivery approaches, we selected two mitochondrion-targeted examples to show the improved potency. In 2018, we published an enrichment-triggered release approach to target CO to the mitochondrion (Figure 8A).^53^ This was achieved through the use of a bimolecular prodrug approach for enrichment in the mitochondrion by conjugation with a cationic triphenyl phosphonium (TPP) moiety. The design takes advantage of the enhanced rate of reaction of biomolecular reactions through enrichment for CO delivery. Readers interested in the chemistry are encouraged to read the original publication. Herein, we highlight the improved potency. Specifically, when the prodrugs were examined in an acetaminophen-induced liver-injury model in mice, it was found that the EC_50_ was at least 10-fold lower for the mitochondrion-targeted prodrug at 0.4 mg/kg than that for similar but nontargeted prodrugs. Cell culture experiments demonstrate the same. Of course, such a targeting approach did not specifically direct the prodrug to the intended organ or site, the liver. Further improvements can be achieved by combining molecular-, organelle-, and biomarker-based targeting. Independently, Berreau et al. reported a mitochondrion-localized visible light triggered CO donors (photoCORMs) containing a TPP moiety (Figure 8B).^295^ The localization of the photoCORMs in the mitochondria was observed by confocal microscopy. Additionally, decreases in ATP production, maximal respiration, and the reserve of A549 cells were also confirmed. The examples described show the potential of further improving the safety margin of CO-based therapeutics through targeted delivery.

## 7 Discussing CO Safety in the Context of Drug Design Is a Different Concept as Compared to Being a Contaminant or Pollutant

With all the discussions of CO’s safety profiles, one might ask the question of how to reconcile with the large body of literature on the undesirable nature of CO in the air as a pollutant^296^ and the possible harmful effects in cigarette smoke.^297−299^ Without getting into the details of the published literature in these two areas, it is important to point out unequivocally that there is a fundamental distinction between safety issues in drug discovery and as a contaminant. Below, we use a few examples to demonstrate this point.

The availability of antibiotics has defined modern-day medical care and has allowed humans to get past the dark ages where minor bacterial infections could lead to lethal consequences. However, the widespread use of antibiotics also presents environmental and health consequences. There have been several findings showing the presence of antibiotics in the water bodies including rivers,^300^ lakes,^301^ seawater,^302^ and most importantly in drinking water.^303^ These antibiotics include sulfonamides, quinolones, tetracyclines (TCs), and β-lactams such as penicillin.^29,300−302,304,305^ Prolonged exposure to these antibiotics at 120 ng/mL (Table 5, entry 2) is considered harmful by some studies.^306^ However, these numbers are far below what has been used for treating bacterial infections. For example, the prescribed dose of ciprofloxacin (a quinolone antibiotic) is 250 mg, leading to blood concentrations of about 3.8 μM.^26^ Such levels are considered safe in the context of treating bacterial infections but would not be considered a safe level of exposure to an otherwise healthy person with no need for such an antibiotic. Another example is the occupational exposure to cytotoxic agents.^307^ Surface exposure of cyclophosphamide at 139 μg/cm^2^ is considered unacceptable for healthcare professionals.^307^ However, this level is much lower than that normally prescribed for treating cancer. For example, cyclophosphamide is normally used at 1–5 mg/kg orally for the treatment of acute lymphocytic leukemia.^308^ There are many other similar examples in Table 5.

**Table 5:** Concentration Comparison of Few Drugs as Pollutants, Prescribed Dosage, and Maximum Dosage^a^

#	drug	threshold concentration as a pollutant (median)	daily unwanted intake of these pollutant^b^	prescribed dosage^c^	maximum dosage^c^	ref
1	ciprofloxacin	270 ng/mL (tap water), 3 ng/mL (rivers)	0.35 mg (tap water), 0.004 mg (rivers)	250 mg every 12 h for three days	1.5 g/day regular release products	(303, 306)
2	azithromycin	120 ng/mL (rivers)	0.156 mg	500 mg on day 1, followed by 250 mg once daily for at least five days	500 mg/day	(306)
3	clarithromycin	235 ng/mL (rivers)	0.305 mg	250 mg every 12 h	1.5 g/day	(306)
4	tetracycline	<23 ng/mL (rivers)	0.030 mg	500 mg twice daily	2 g/day	(306)
5	trimethoprim	424 ng/mL (rivers)	0.551 mg	100 mg every 12 h	200 mg/day	(306)
6	venlafaxine	395 ng/mL (rivers)	0.513 mg	75 mg/day divided in two or three doses	375 mg/day	(306)
7	carbamazepine	455 ng/mL (rivers)	0.591 mg	200 mg twice daily	1200–1600 mg/day	(306)
8	caffeine	1898 ng/mL (rivers)	2.47 mg	100 to 200 mg, may repeat dose every 3–4 h	1200 mg/day	(306)

a The exposure levels and daily unwanted exposure might differ depending on which reference is checked. These values are not meant as guidelines.

b Estimated based on daily water consumption, 44 ounces.^309^

c Extracted from a physician desk reference.^308^ Concentrations are calculated and presented here for a comparative study.

All of these examples emphasize the point that the concepts and contexts are different when considering the development of a therapeutic as compared to a pollutant/contaminant. By the same token, even if CO is eventually approved for treating a disease, a healthy person is not meant to be exposed to it for no specific reasons and justifications. This is the same as saying that people who do not have cancer do not need to be exposed to anticancer agents, and people who have no viral infections do not need to be exposed to antivirals. The risk analysis is different when considering a chemical as a therapeutic agent as compared with being a contaminant/pollutant. Therefore, the available data on CO’s harmful effects in the air or in cigarette smoke are actually not incongruent with the use of CO as a therapeutic agent.

## 8 Reversible Binding and the Issue of Chronic Effects

With all the discussions in the previous sections, it is clear that there is a wide operational range of COHb levels for therapeutic applications of CO. There is one additional question, i.e., chronic toxicity, or cumulative effects at low levels. Since there have never been extensive studies of this subject at the molecular level, we can only provide some theoretical analyses of this issue. Along this line, one can think of exposure to slightly elevated levels of glucose (diabetes), which causes chronic health problems, and heavy metals, which cause chronic and cumulative toxicity issues even with low levels of exposure. Herein, we analyze the issue in the context of the volatility of CO and the reversible nature of the binding of CO to its targets.

First of all, the endogenous production as part of normal physiology should already indicate the lack of toxicity long-term at or below 2% COHb.^20^ We feel that it is indeed true that chronic exposure to endogenous and physiological levels of CO should not present toxicity issues. It is well-known that various saccharides including glucose can covalently modify proteins.^310^ One might ask the question of glucose’ ability to glycate proteins as an indication that “physiological” molecules could present chronic toxicity issues. Indeed, glucose has a narrow safety margin, as discussed earlier. Diabetic conditions with a slightly elevated level of glucose could lead to an increased level of glycated proteins, which results from the reaction of the glucose molecule (aldehyde group) with an amino group of biomolecules as well as subsequent rearrangements. Such reactions result in covalent modifications of proteins, leading to long-term toxicity issues. This is part of the reason that glycate hemoglobin fraction A1C is used as a surrogate marker for monitoring sustained glucose levels over a period of 3 months in diabetic patients.^310^ If glucose can cause chronic toxicity, why not other “physiological” molecules? Indeed, one cannot say for sure unless there are clinical data. Further, this is a very difficult subject on which to truly conduct carefully controlled studies. However, one can probably draw two conclusions. First, the reversible nature of the binding of CO to its targets is in direct contrast to the ability of glucose to covalently modify proteins and possible other biomolecules. Without irreversible covalent modification, one would not expect cumulative effects the same way as for protein glycation. Second, the lack of irreversible modification of biomolecules likely means that CO is not expected to have the same level of chronic toxicity issue as glucose. If CO is “safer” in the context of safety margin and chronic toxicity compared to glucose, it is a reassuring position for therapeutic applications, even if it is not “safe” in an absolute sense.

After the comparison with glucose, one could also think of heavy metal toxicity upon exposure at low levels. It is important to note that most metal binding to key functional groups in biomolecules is also reversible, even if it is not as readily reversible as CO binding to a hemoprotein. Then, how does one compare the effect on chronic and cumulative toxicity due to metal binding or CO binding, since both are reversible? For example, when heavy metals are discharged with wastewater, even if the concentration is very low, they can accumulate in algae and sediment and be adsorbed by fish or other aquatic creatures, causing harm to the upper levels of the food chain. Being at the top of the food chain, humans end up being exposed to all of the accumulated heavy metals (e.g., cadmium, lead, mercury, and arsenics) upstream of the food chain. These metal ions are known to interact with proteins and various enzymes, modifying or inactivating their functions.^311,312^ For instance, cadmium is known to have the ability to displace Mg, Zn, and Ca ions in some proteins (e.g., calmodulin and troponin C) because of its similar biophysical and chemical properties.^313^ In 1984, Ellis et al. measured cadmium’s binding affinity to skeletal troponin C (STnC) by ^113^Cd NMR spectroscopy and found a similar affinity compared to that of Ca and Mg (*K*_Cd_ = 10^7^ M^–1^).^314^ Itai-itai disease is an example of cadmium exposure. Lead (Pb) also can interact with proteins that have a bound calcium, such as calmodulin, protein kinase C, and synaptotagmin I. In 1988, Markovac et al. found the capability for a low level of Pb (10^–10^ M^–1^) to activate protein kinase C to the same extent as micromolar calcium. Pb has also been shown to affect different types (N-, L-, and T-type) of voltage-activated calcium channels when studied using N1E-115 mouse neuroblastoma cells. Pb has been found to block calcium channels at nanomolar to micromolar concentrations. As a brief summary, both Cd(II) and Pb(II) can form complexes with the S and N donors from proteins (Cys, Glu, and His), hindering the functions of the native ions such as Zn, Ca, and Mg.^313,315^ Mercury is well-known for its toxicity in all three forms including metallic, mercuric (Hg(II)), and organic mercury. Hg(II) has a similarity with Cd(II) and Pb(II) in its affinity with thiol species and thus toxicity. Moreover, HgCl_2_ was also widely used as an important ingredient in many skin-lightening products. Hg^2+^ is known to irreversibly inhibit tyrosinase by replacing the copper cofactor with a inhibition constant value (*K*_i_) of 29.4 μM.^316^ Because of its toxicity, a global agreement “Minamata Convention” was reached in 2017 banning the manufacture, import, or export of skin lightening soaps with a mercury content higher than 1 ppm after 2020.^317^ Organic mercury compounds (methyl mercury as the majority) are more toxic than inorganic forms; humans usually get it from fish, and it accumulates in the body. Many diseases show a correlation with organic mercury, such as Minamata disease, hypertension, cardiovascular disease, and stroke.^318^ Arsenic toxicity is mainly from the inorganic arsenite(III) and arsenate(V). Arsenite(III) can conjugate with GSH, leading to altered protein functions. Arsenite(III) is also known to contribute to the production of reactive oxygen and nitrogen species, leading to damage of biomacromolecules. Arsenate(V) has a similar structure to phosphate, leading to phosphate replacement in the glycolysis and decreased production of ATP.^319^

In an analysis of this matter, it is important to note a major distinction: CO is volatile, and heavy metals are not. Further, the tight binding between a heavy metal ion and biomacromolecule components contributes to its long-term retention. The difference in retention time contributes to a key difference in the cumulative effects of both. For example, the biological *t*_1/2_ of methylmercury has been reported to range from 30 to 120 days (average 50 days).^320^ However, in the brain it has been estimated to be as long as 20 years.^321^ This gives a chance for cumulative effects. For cadmium, the biological *t*_1/2_ was estimated to be 6–38 years in the kidney and 4–19 years in the liver.^322^ For lead, the biological *t*_1/2_ has been reported to be 40 days in the blood.^323^ The COHb dissociation kinetics varied among the different species. For mice, the reported half-life of COHb is around 20 min.^267,324^ However, for humans, the half-life is around 3–4 h.^187^ Further, CO binding to hemoproteins can be reversed by oxygen or hyperbaric oxygen when severe CO poisoning is involved. All these examples indicate that CO does not have a long-term accumulation issue and its binding to hemoproteins is also reversible.

As a summary, CO’s volatility, the reversible nature of its binding, and its relatively short half-life in humans all mean that it does not accumulate the same way as heavy metals or engage in covalent interactions the same way as organic molecules with electrophiles such as glucose (aldehyde).

## 9 Conclusion

In this Perspective, we have focused on the question of whether CO is safe enough for therapeutic applications. The safety profiles of CO have been shown by considering the following factors: (1) endogenous production of CO and its concentrations under various pathophysiological conditions; (2) its safety margin in comparison to commonly used drugs, other endogenous bioactive molecules, and even nutrients; (3) the anticipated enhanced safety profiles when delivered via a noninhalation mode; (4) the anticipated enhanced safety profiles via targeted delivery; and (5) the large amount of safety data from human clinical trials. We have provided evidence and critical analyses to show that CO exhibits a safety margin comparable to or wider than those of various endogenous bioactive molecules, nutrients, and FDA approved drugs. We demonstrate that noninhalation routes are safer than the widely used inhalation route. We have also proposed a detailed mechanism to account for the difference in safety profiles when the delivery route is different. A corollary question is whether it is easier to deliver CO to tissues using HB(CO)_4_ than HB(CO)(O_2_)_3_. Along this line, we have devoted major efforts to developing noninhalation CO delivery forms, which is another topic. It is also important to note that the critical analysis of the safety profiles of CO simultaneously demonstrates a sufficiently high safety margin for CO as a potential drug and is consistent with the known harmful effects of CO as a pollutant or contaminant. This is because of the clear distinction in safety analysis between the context of CO being used to treat a health problem and the context of it being considered as a contaminant. These two issues are indeed very different. At this point, it is important to note that the numbers quoted in the tables are meant as references for research purposes and not safety guidelines. Further, some of these specific numbers vary depending on which publications are read. At the end, we hope this Perspective will stimulate similar discussions and research in understanding the pharmacological and toxic effects of CO, including associated mechanism(s) of actions.

## Abbreviations Used

CO: Carbon monoxide
COHb: Carboxyhemoglobin
IUPAC: International Union of Pure and Applied Chemistry
HMOX: Heme oxygenase
GI: Gastrointestinal
HMOX-1: Heme oxygenase-1
HMOX-2: Heme oxygenase-2
PK: Pharmacokinetic
EPA: U.S. Environmental Protection Agency
FDA: U.S. Food and Drug Administration
ETCO: End tidal carbon monoxide
NO: Nitric oxide
OphVB: Ophthalmic venous blood
2,3-DPG: 2,3-Diphosphoglycerate
FMD: Flow-mediated dilation
COPD: Chronic obstructive pulmonary disease
PKU: Phenylketonuria
INR: International normalized ratio
PTSD: Post-traumatic stress disorder
Mb: Myoglobin
CcO: Cytochrome c oxidase
*P*^O2^: Partial pressure of oxygen
O_2_Hb: Oxyhemoglobin
*K*_a_: Association constant
*K*_d_: Dissociation constant
*k*_on_: Association rate constant
*k*_off_: Dissociation rate constant
CORM: CO-releasing molecules
TPP: Triphenyl phosphonium
EC_50_: Half-maximal effective concentration
TCs: Tetracyclines
STnC: Skeletal troponin C
*K*_i_: Inhibition constant
